# Benzyl-Naphthoquinones as Selective Anticancer Agents for Oral Squamous Cell Carcinoma via Apoptosis Induction

**DOI:** 10.3390/biomedicines14040757

**Published:** 2026-03-26

**Authors:** Antonio Mendonça Marconi-Nicolau, Rebeca Gripp de Sá, Caroline Reis Santiago Paschoal, Lethícia Andrade de Almeida, Gabriel Ouverney, Ana Caroline dos Santos-Diniz, Anamel Blaudt Meira, João Pedro da Costa Faria Brunhosa, Luiz Carlos da Silva Pinheiro, Paula Alvarez Abreu, Vinicius Rangel Campos, Bruno Kaufmann Robbs

**Affiliations:** 1 Programa de Pós-Graduação em Ciências Morfológicas, Instituto de Ciências Biomédicas, Universidade Federal do Rio de Janeiro, Fundão, Rio de Janeiro 21941-902, RJ, Brazil; antoniomarconi@id.uff.br (A.M.M.-N.); ouverneygabriel@id.uff.br (G.O.); 2 Institute of Chemistry, Department of Organic Chemistry, Campus do Valonguinho, Universidade Federal Fluminense, Niterói 24020-150, RJ, Brazil; rebecagrippdesa@gmail.com (R.G.d.S.); lethicia.andrade2@gmail.com (L.A.d.A.); joaopedrobrunhosa@id.uff.br (J.P.d.C.F.B.); 3 Instituto de Biodiversidade e Sustentabilidade NUPEM, Universidade Federal do Rio de Janeiro, Macaé 27965-045, RJ, Brazil; carolinesantiago@live.com (C.R.S.P.); abreu_pa@yahoo.com.br (P.A.A.); 4 Nova Friburgo Health Institute, Department of Basic Sciences, Universidade Federal Fluminense, Nova Friburgo 28625-650, RJ, Brazil; ana_diniz@id.uff.br (A.C.d.S.-D.); anamelmeira@gmail.com (A.B.M.); 5 Faculdade de Formação de Professores, Departamento de Ciências, Universidade do Estado do Rio de Janeiro—UERJ, São Gonçalo 24435-005, RJ, Brazil; pinheirolcs@gmail.com

**Keywords:** oral squamous cell carcinoma, naphthoquinones, selectivity, cytotoxicity, chemotherapy

## Abstract

**Background**: Oral squamous cell carcinoma (OSCC) is an aggressive cancer closely associated with smoking and alcohol consumption, with a higher incidence in men. Despite changes in treatment strategies, poor survival persists in most patients, highlighting the need for novel and improved therapeutic options. Naphthoquinone analogs are being investigated because of their active redox structure and broad pharmacological profile; they demonstrate cytotoxic antitumor activity, making them potential candidates for new drug agents. **Objective**: This study investigated new benzyl-naphthoquinone compounds as potential anticancer agents for various genotypes of oral squamous cell carcinoma (OSCC) and other cancer cells. **Methods**: This study reports the synthesis and evaluation of a series of eight benzyl-naphthoquinone compounds against oral squamous cell carcinoma. **Results**: Four compounds **1**–**4** showed the best cytotoxic profiles, with a selectivity index ≥ 3 for all OSCC cell lines tested. Compound **1** was the most selective compound in all OSCC models, showing a higher selectivity index than both carboplatin and shikonin. Furthermore, compound **1** induced DNA fragmentation, cell-cycle arrest, and caspase-3/7 activation, changes consistent with apoptosis, and time-lapse imaging corroborated the apoptotic phenotype. Hemolysis assays showed minimal toxicity in human erythrocytes, and acute in vivo evaluation in mice revealed no evident adverse effects under the conditions tested, indicating low acute toxicity, although more detailed histopathological and biochemical studies will be required to fully establish the safety profile. Molecular modeling suggested that compound **1** may interact with topoisomerase II, RSK2, and PKM2, which could contribute to the activation of apoptotic pathways, although these interactions remain predictive and require biochemical validation. Finally, in silico analysis of physicochemical and ADMET parameters indicated properties compatible with oral absorption and systemic exposure, together with predicted low toxicity; however, these results are model-based and should be confirmed experimentally. **Conclusions**: Based on these findings, compound **1** emerges as a promising lead candidate for the development of a novel chemotherapeutic agent against OSCC, with potential therapeutic efficacy against other cancer types.

## 1. Introduction

Oral squamous cell carcinoma (OSCC) accounts for more than 90% of oral malignancies and remains a major global health challenge owing to its high incidence, aggressive progression, and low survival rates [1]. Although there has been progress in surgery, radiotherapy, and chemotherapy, the prognosis for OSCC has not significantly improved over the past few decades, with survival rates remaining below 50% in advanced stages [2]. Conventional chemotherapeutic agents, such as platinum-based compounds, are widely used in clinical practice. However, they are often associated with severe systemic toxicity, limited selectivity for tumor cells, and the development of drug resistance [3]. These limitations highlight the need for safer and more effective therapeutic alternatives that selectively target cancer cells while sparing healthy tissues.

Natural products and their compounds have long been recognized as valuable sources of bioactive molecules for the development of anticancer drugs. Among them, naphthoquinones have attracted considerable attention because of their broad pharmacological activities, including the induction of apoptosis, generation of reactive oxygen species (ROS), and inhibition of tumor growth in various cancer models [4]. Shikonin, a naturally occurring naphthoquinone, has demonstrated potent antitumor effects through multiple mechanisms, including the induction of apoptosis and autophagy, modulation of reactive oxygen species, and inhibition of key survival signaling pathways. These pleiotropic effects contribute to their ability to suppress tumor cell proliferation and promote cancer cell death across diverse tumor models [5]. These findings have stimulated the rational design and synthesis of novel naphthoquinone derivatives with improved pharmacological properties. Naphthoquinones are recognized as important pharmacophores/privileged structures [6] in the search for new anticancer agents, as they exhibit diverse biological activities and allow extensive structural modifications within their core scaffold [7]. In particular, based on the well-documented antitumor activity of naturally occurring naphthoquinones such as shikonin, structural optimization strategies, such as the introduction of benzyl moieties, have been explored to enhance efficacy, selectivity, and lipophilicity of these synthetic compounds.

**Figure 1:**
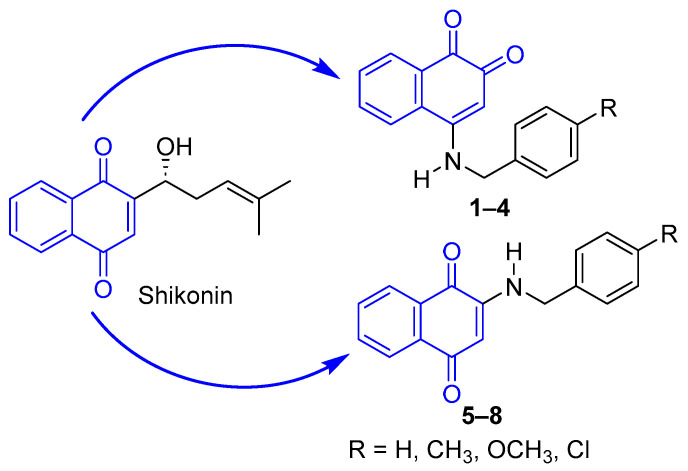
Rational approach to the design of compounds **1**–**8**.

In this study, we report the synthesis and biological evaluation of eight benzyl-naphthoquinone compounds **1**–**8** from shikonin (Figure 1). Among these eight benzyl-naphthoquinones, three (**2**–**4**) have not been previously described. This investigation explored the potential mechanism of action and evaluated the pharmacokinetic and toxicity profiles of the most promising compound using a combination of in silico, in vitro, and in vivo approaches to assess its viability as a candidate for developing a novel treatment for OSCC.

## 2. Materials and Methods

### 2.1. Chemistry

#### 2.1.1. General Experimental Procedures

The 4-benzylamine-1,2-naphthoquinones **1**–**4** and 2-benzylamine-1,4-naphthoquinones **5**–**8** were obtained from the reaction between sodium 1,2-naphthoquinone-4-sulfonate **(9)** or naphthalene-1,4-dione **(10)**, respectively, and the respective benzylamines under ultrasound irradiation at room temperature for 1 h (Scheme 1). All chemical compound spectra are at Supplementary data (Supplementary Figures S1–S25).

All reagents and solvents used were of analytical grade. TLC was performed using silica gel Merck (Darmstadt, Germany) TLC F-254 PTLC (preparative TLC, ) glass plates (20 × 20 cm). The melting points (m.p.s) were determined using a Fisatom (São Paulo, Brazil) model 430D apparatus. High-resolution mass spectrometry (HRMS) data were obtained using an LC-MS Bruker Daltonics MicroTOF (time-of-flight) analyzer (Billerica, MA, USA). Fourier-transform infrared (FTIR) absorption spectra were recorded on a Nicolet iS50 FT-IR—Thermo Scientific spectrophotometer (Waltham, MA, USA) using the attenuated total reflection (ATR) technique. ^1^H and ^13^C nuclear magnetic resonance (NMR) spectra were obtained at 500.00 and 125.00 MHz, respectively, using a Bruker (Billerica, MA, USA) Avance instrument with a 5 mm probe. Tetramethylsilane was used as an internal standard. The chemical shifts (δ) are reported in ppm and the coupling constants (J) are reported in Hertz (Hz).

#### 2.1.2. Purity Assessment of Synthesized Compounds

The purity of the synthesized compounds was evaluated based on the combined analysis of melting point determination, IR spectroscopy, ^1^H NMR, ^13^C NMR, and high-resolution mass spectrometry (HRMS). The ^1^H and ^13^C NMR spectra showed clean baselines and signal integrations consistent with the proposed structures, with no detectable extraneous peaks corresponding to impurities or residual starting materials. The compounds also exhibited sharp melting point ranges (≤2 °C), supporting sample homogeneity. HRMS analysis confirmed the exact molecular masses with high precision. Together, these orthogonal analytical techniques support the high purity of the isolated compounds used in the biological assays. These analytical criteria have been routinely employed in previously published studies involving related compounds and have been accepted as evidence of compound purity in several publications [8,9,10,11].

#### 2.1.3. Procedure for Preparing 4-Benzylamine-naphthalene-1,2-dione 1-4 and 2-Benzylamine-naphthalene-1,4-dione 5-8

A mixture of sodium 1,2-naphthoquinone-4-sulfonate (**9**) 165 mg (0.6323 mmol) or naphthalene-1,4-dione (**10**) 100 mg (0.6323 mmol) and the appropriate amine: phenylmethanamine; 4-tolylmethanamine; (4-methoxyphenyl)methanamine and (4-chlorophenyl)methanamine (0.6323 mmol) in 5 mL of EtOH and 5 mL of H_2_O were stirred at 25 °C for 1 h, under ultrasound irradiation. After the end of the reaction, as evidenced by TLC using a chloroform:methanol (9:1) elution system, the precipitate formed was vacuum-filtered and washed with water. The residual crude product was purified by silica gel column chromatography using a gradient mixture of hexane/ethyl acetate (7:3). Compounds **1**–**8** were obtained as orange solids with 15–70% yield.

4-(benzylamino)naphthalene-1,2-dione (**1**). Yield: 25%. m.p.: 195–196 °C. IR (cm^−1^): 3243; 1685; 1585; 738. ^1^H NMR (500 MHz, CDCl3, TMS, *δ* in ppm): 4.62 (s, 1H); 5.61 (s, 1H); 7.34–7.24 (m, 4H); 8.10–7.66 (m, 5H). HMRS (ESI): calculated for C_17_H_13_NO_2_ 286.084; found [M + Na]^+^ 286.0841 [12].

4-((4-methylbenzyl)amino)naphthalene-1,2-dione (**2**). Yield: 15%. m.p.: 218–219 °C. IR (cm^−1^): 3360; 1673; 1603; 1259. ^1^H NMR (500 MHz, DMSO-d6, TMS, *δ* in ppm): 2.28 (s, 3H); 4.58 (d, *J* = 5.9 Hz, 1H); 5.60 (s, 1H); 7.17 (d, *J* = 8.0 Hz, 2H); 7.27 (d, *J* = 8.0 Hz, 2H); 8.18–7.67 (m, 4H); 8.83 (s, 1H). ^13^C NMR (125 MHz, DMSO-d6, TMS, δ in ppm): 20.5; 46.1; 98.9; 123.2; 126.9; 127.7; 129.0; 129.4; 130.8; 131.1; 134.1; 134.1; 136.4; 154.9; 175.3; 181.7. HMRS (ESI): calculated for C_18_H_15_NO_2_ 300.100; found [M + Na]^+^ 300.0981.

4-((4-methoxybenzyl)amino)naphthalene-1,2-dione (**3**). Yield: 40%. m.p.: 223–224 °C. IR (cm^−1^): 3330; 1697; 1586; 1240. ^1^H NMR (500 MHz, MeOD, TMS, *δ* in ppm): 3.78 (s, 3H); 4.66 (s, 2H); 5.87 (s, 1H); 6.93 (d, *J* = 8.78 Hz, 2H); 7.31 (d, *J* = 8.78 Hz, 2H); 7.72–7.68 (m, 1H); 7.82–7.79 (m, 1H); 8.06–8.05 (m, 1H); 8.13–8.11 (m, 1H). HMRS (ESI): calc. for C_18_H_15_NNaO_3_ 316.0950; found [M + Na]^+^ 316.0947.

4-((4-chlorobenzyl)amino)naphthalene-1,2-dione (**4**). Yield: 20%. m.p.: 226–227 °C. IR (cm^−1^): 3322; 1698; 1586; 1541; 838. ^1^H NMR (500 MHz, DMSO-d6, TMS, *δ* in ppm): 4.60 (s, 2H); 5.57 (s, 1H); 7.37 (s, 4H); 7.68 (td, *J* = 7.5, 0.7 Hz, 1H); 7.80 (td, *J* = 7.7, 1.4 Hz, 1H); 7.97 (dd, *J* = 7.6, 1.3 Hz, 1H); 8.08–8.06 (m, 2H). HMRS (ESI): calc. for C_17_H_12_ClNNaO_2_ 320.0454; found [M + Na]^+^ 320.0444.

2-(benzylamino)naphthalene-1,4-dione (**5**) Yield: 46%. m.p.: 158–160 °C. IR (cm^−1^): 3332; 1681. ^1^H NMR (500 MHz, CDCl3, TMS, *δ* in ppm): 4.45 (d, *J* = 6.5 Hz, 2H); 5.59 (s, ^1^H); 7.28–7.23 (m, 1H); 7.38–7.31 (m, 4H); 7.72 (td, *J* = 7.5, 1.3 Hz, 1H); 7.80 (td, *J* = 7.5, 1.3 Hz, 1H); 7.91 (dd, *J* = 7.8, 1.0 Hz, 1H); 7.95 (s, 1H); 8.00 (dd, *J* = 7.7, 0.9 Hz, 1H). HMRS (ESI): calculated for C_17_H_13_NNaO_2_ 286.0844; found [M + Na]^+^ 286.0831 [13,14].

2-((4-methylbenzyl)amino)naphthalene-1,4-dione (**6**). Yield: 30%. m.p.: 169–171 °C. IR (cm^−1^): 3360; 1673. ^1^H NMR (500 MHz, CDCl3, TMS, *δ* in ppm): 2.35 (s, 3H); 4.33 (d, *J* = 5.7 Hz, 2H); 5.79 (s, 1H); 6.17 (s, 1H); 7.18 (d, *J* = 8.1 Hz, 2H); 7.21 (d, *J* = 8.2 Hz, 2H); 7.62 (td, *J* = 7.6, 1.3 Hz, 1H); 7.72 (td, *J* = 7.6, 1.3 Hz, 1H); 8.05 (dd, *J* = 7.7, 0.9 Hz, 1H); 8.09 (dd, *J* = 7.7, 0.9 Hz, 1H). HMRS (ESI): calculated for C_18_H_15_NNaO_2_ 300.1000; found [M + Na]^+^ 300.0977 [15,16].

2-((4-methoxybenzyl)amino)naphthalene-1,4-dione (**7**). Yield: 50%. m.p.: 165–166 °C. IR (cm^−1^): 3332; 1681. ^1^H NMR (500 MHz, CDCl_3_, TMS, *δ* in ppm): 3.81 (s, 3H); 4.31 (d, *J* = 3.8 Hz, 2H); 5.84 (s, 1H); 6.18 (s, 1H); 6.90 (d, *J* = 8.7 Hz, 2H), 7.24 (d, *J* = 8.7 Hz, 2H); 7.62 (td, *J* = 7.5, 1.2 Hz, 1H); 7.72 (td, *J* = 7.6, 1.3 Hz, 1H); 8.05 (dd, *J* = 7.7, 1.0 Hz, 1H); 8.09 (dd, *J* = 7.7, 0.9 Hz, 1H). HMRS (ESI): calculated for C_18_H_15_NNaO_3_ 316.0950; found [M + Na]^+^ 316.0944 [14,16].

2-((4-chlorobenzyl)amino)naphthalene-1,4-dione (**8**). Yield: 70%. m.p.: 213–215 °C. IR (cm^−1^): 3355; 1671. ^1^H NMR (500 MHz, CDCl_3_, TMS, *δ* in ppm): 4.37 (d, *J* = 5.8 Hz, 2H); 5.75 (s, 1H); 6.18 (s, 1H); 7.25 (d, *J* = 7.4 Hz, 6H); 7.35 (d, *J* = 8.4 Hz, 2H); 7.63 (td, *J* = 7.6, 1.2 Hz, 1H); 7.74 (td, *J* = 7.6, 1.2 Hz, 1H); 8.07 (d, *J* = 7.6 Hz, 1H); 8.09 (d, *J* = 7.7 Hz, 1H). HMRS (ESI): calculated for C_17_H_12_ClNNaO_2_ 320.0454; found [M + Na]^+^ 320.0449 [15,16].

### 2.2. Biological Assays

#### 2.2.1. Cell Lines and Culture Conditions

Human cancer cell lines with different origins as oral squamous cell carcinomas (SCC-4, SCC-9, and SCC-25), hepatocarcinoma (HepG2), colon carcinoma (HT-29, HCT-116), cervical carcinoma (HeLa), murine melanoma (B16-F10), and murine breast carcinoma (4T1) were obtained from the American Type Culture Collection (ATCC, Manassas, VA, USA) (ATCC numbers: CRL-1624, CRL-1629, CRL-1628, HB-8065, HTB-38, CCL-247, CCL-2, CRL-6475, CRL-2539, respectively). Primary normal human gingival fibroblasts (HGFs) were obtained from ATCC (PCS-201-018). SCCs were maintained in 1:1 DMEM/F12 (Dulbecco’s modified Eagle medium (DMEM) and Ham’s F12 medium (Gibco; Thermo Fisher, Waltham, MA, USA) supplemented with 10% (*v*/*v*) FBS (fetal bovine serum (FBS; Invitrogen, Thermo Fisher, Waltham, MA, USA) and 400 ng/mL hydrocortisone (Sigma-Aldrich Co., St. Louis, MO, USA), whereas other cell lines were maintained in DMEM supplemented with 10% (*v*/*v*) FBS. Cells were grown in a humidified environment containing 5% CO_2_ at 37 °C. For all biological experiments, the tested compounds and control menadione were solubilized in 100% DMSO (Sigma-Aldrich Co., St. Louis, MO, USA) to a final concentration of 10 mM. Chemotherapeutical agent carboplatin stock was commercially acquired (Fauldcarbo^®^; Libbs Farmacêutica, São Paulo, SP, Brazil) and shikonin (Sigma-Aldrich Co., St. Louis, MO, USA) was used as a standard anticancer compound.

#### 2.2.2. MTT Cytotoxicity Assay

Cell viability was assessed using the MTT assay, as previously described [17]. Cells were seeded at a density of 5 × 10^3^ cells/well in 96-well plates and allowed to adhere for 24 h. The cells were then exposed to different concentrations of naphthoquinone compounds (up to 200 µM) for 48 h. Shikonin (≤25 µM) and carboplatin (≤800 µM) were used as reference drugs, whereas untreated cells and vehicle (DMSO at the same concentrations was used as a 100% cell viability standard) served as controls. After treatment, MTT reagent (0.5 mg/mL) was added and incubated for 3.5 h. The tetrazolium salt (MTT) is reduced by mitochondrial dehydrogenases of metabolically active cells to purple formazan crystals, which accumulate inside viable cells. Formazan crystals were solubilized with a DMSO/methanol solution, and absorbance was measured at 560 nm using an EPOCH microplate spectrophotometer (BioTek Instruments, Winooski, VT, USA). Cytotoxicity assays were performed in three independent experiments, each conducted using different passages of the same cell line. In each experiment, cells were freshly plated and the tested compounds were independently resuspended and prepared, characterizing biological replicates. Technical replicates were performed within each experiment as described above.

#### 2.2.3. Statistical Analysis

Half maximal inhibitory concentration (IC_50_) values were obtained from the MTT assay of at least three independent replicates through non-linear regression analysis using GraphPad Prism 5.0 software (Intuitive Software for Science, San Diego, CA, USA). Data were presented as means ± standard deviation (SD). A log dose–response curve (inhibitor vs. response) using the least squares method was used to determine the IC_50_ and SD from the data. The selectivity index (SI) was calculated as the ratio between the IC_50_ in normal cells and the IC_50_ in cancer cells, obtained by dividing the IC_50_ of normal cells by the IC_50_ of cancer cells.

#### 2.2.4. Hemolysis Assay

The hemolytic potential of the compounds was determined following established protocols [18]. Fresh human erythrocytes were isolated and incubated with different concentrations of naphthoquinone compounds and were approved by the Research Ethics Committee of Universidade Federal Fluminense (CAAE: 43134721.4.0000.5626; approval date of the study 18 April 2024). Signed informed consent forms for all participants were obtained. Triton X-100 was used as the positive control (100% hemolysis), while PBS served as the negative control. Hemoglobin release was quantified by measuring absorbance at 540 nm with EPOCH (BioTek Instruments, Winooski, VT, USA).

#### 2.2.5. Time-Lapse Assay

SCC9 cells were seeded in 35 mm culture dishes one day before the experiment to allow cell adhesion and stabilization as previously described [9]. Cells were then treated with 2 × IC_50_ of compound **1** (4.72 µM) or with DMSO (vehicle control) and immediately transferred to a culture chamber coupled to a Nikon Eclipse TE300 inverted microscope (Nikon, Melville, NY, USA), maintained under controlled conditions of 5% CO_2_ and 37 °C. Phase-contrast images of a fixed field were acquired at 1 min intervals for a total period of 48 h. The image sequences obtained for each condition were compiled into time-lapse videos using ImageJ software (v. 1.54f; National Institutes of Health, USA). Specific time points were extracted to illustrate the morphological changes observed throughout the treatment.

#### 2.2.6. Caspase 3/7 Activity Assay

Caspase activity was evaluated using the Caspase-Glo^®^ 3/7 assay kit (Promega, Madison, WI, USA) according to the manufacturer’s instructions. SCC9 cells were treated with 2 × IC_50_ (4.72 µM) of compound **1**, and luminescence was measured using a TD 20/20 luminometer (Turner Designs, Sunnyvale, CA, USA). The reading of bioluminescence detected is indicated as arbitrary units (a.u.). Results were normalized to untreated controls.

#### 2.2.7. DNA Fragmentation and Cell Cycle Analysis

To evaluate the action of compound **1** on the cell cycle and DNA fragmentation, cells of the SCC9 cell line were plated in a 6-well plate (5 × 10^5^ cells/well) and treated with 2 × IC_50_ (4.72 µM) of compound **1** or with DMSO (vehicle control). After 24 (cell cycle) or 48 h (DNA fragmentation) of treatment, cells were trypsinized and stained with propidium iodide (75 µM) in the presence of NP-40. DNA content was analyzed by collecting 10,000 events using a FACScalibur flow cytometer (Becton Dickinson, San Jose, CA, USA). Data were analyzed using flow cytometry analysis using cytometer Guava^®^ Muse Cell Analyzer (Cytek Biosciences, Fremont, CA, USA) and Flowing Software (v. 2.5.1; Turku Biosciences, Turku, Finland).

#### 2.2.8. Autophagy Marker Analysis by Fluorescence Microscopy

To determine whether compound **1** induces autophagy, SCC-9 cells were stably transduced with a plasmid expressing LC3-GFP as described previously [19]. Briefly, 2.5 × 10^4^ SCC-9-LC3-GFP cells were plated in 500 µL of medium per well in 24-well plates and, after 24 h, treated with compound **1** at its IC_50_ concentration (2.36 µM) or DMSO and incubated. As a positive control, we used Couma.6e (Supplementary Figure S26), a previously described naphthoquinone–triazole–coumarin hybrid capable of inducing autophagy [20] at its IC_50_ concentration, 30 µM. Cells were analyzed for GFP–LC3 puncta formation at 6, 12, 24, 36, and 48 h after treatment. As compound **1** did not induce detectable puncta formation at any evaluated time point, the 36 h time point, at which the positive control displayed the most evident staining [20], was selected for representative analysis and image presentation.

The experiment was performed in duplicate in three independent experiments. For each experiment, 10 images of randomly selected fields were acquired at 20× magnification using a Zeiss Axio Observer A1 fluorescence microscope (Zeiss, Oberkochen, Baden-Württemberg, Germany). A minimum of 100 cells per experiment were analyzed. Quantification was performed using ImageJ software (National Institutes of Health, Bethesda, MD, USA). Cells were classified as positive for autophagy when presenting more than five clearly visible GFP–LC3 puncta. The percentage of puncta-positive and puncta-negative cells was then calculated.

#### 2.2.9. In Vivo Acute Toxicity Study

The acute toxicity of compound **1** was assessed following ethical guidelines and approved by the Federal Fluminense University Animal Ethics Board (registration number 2699110419; approval date of the study 16 May 2025), and all procedures complied with Brazilian regulations and OECD guideline 423. Twelve-week-old female C57BL/6 mice were used, with three animals per group. Each mouse received a single intraperitoneal injection of compound **1** dissolved in PBS containing 3% DMSO. Control animals received PBS with 3% DMSO only. The compound was given in escalating doses: 200 mg/kg, 400 mg/kg, and 800 mg/kg, based on the responses observed at lower doses. Animals were monitored daily for mortality, toxic effects, and changes in food or water consumption. At the end of the observation period, major organs were collected and examined macroscopically. Across all doses, including 800 mg/kg, no significant mortality or overt toxicity was observed, supporting further preclinical evaluation.

### 2.3. In Silico Studies

#### 2.3.1. In Silico ADMET Prediction

The pharmacokinetic and toxicity profiles of the most propitious compound, compound **1**, were computationally predicted and compared with those of carboplatin and shikonin using the ADMETsar 3.0 (http://lmmd.ecust.edu.cn/admetsar3; accessed on 27 June 2025). The compound’s SMILES structure was generated and submitted to the platform for in silico prediction of key parameters of drug-likeness assessment based on Lipinski’s rule of five.

#### 2.3.2. Molecular Docking Studies

Molecular docking studies were carried out to identify a potential target for 1. Initially, the structure of 1,2-naphthoquinone was constructed using Spartan’10 software for Windows 2011 (Wavefunction Inc., Irvine, CA, USA). A conformational search was performed using the MMFF force field, with the lowest energy conformer being optimized using the PM3 semiempirical method. A single-point electronic structure calculation was then performed using the Hartree–Fock method with the 6-31-G* basis set on the PM3-optimized geometry in order to compute the molecular electrostatic potential. Partial atomic charges were derived from this potential using electrostatic charges, and these charges were employed in subsequent docking calculations for the ligand. Some naphthoquinones with anticancer activity and effects on different targets were subjected to the same protocol for comparative purposes. Thus, the naphthoquinones used as a reference for the investigated binding mode were lapachol, which targets RSK2 and PKM2; shikonin, which targets PKM2; 1,4-naphthoquinone, with inhibitory action on the ATPase domain of topoisomerase IIα; and doxorubicin, which acts on the DNA-binding domain of topoisomerases I, IIα, and IIβ. All compounds were prepared using the same protocol, including electrostatic charge assignments. To perform molecular docking, solvents and artifacts present in the protein’s structures were removed, and polar hydrogens and Gasteiger charges were added. The proteins were kept rigid, and the ligands were maintained flexible, with their total rotatable bonds defined automatically. All calculations were performed with Autodock Tools 1.5.7 and Autodock Vina 1.1.2 software. Molecular redocking of all ligands co-crystallized in the active site of the receptors was performed in order to validate the molecular docking parameters (Table 1) [21,22,23]. The pose with the lowest binding energy for each compound obtained in the docking studies was visually verified, and its interactions were analyzed using Discovery Studio Visualizer 2019 (Dassault Systèmes BIOVIA, San Diego, CA, USA, 2019) and PyMOL v. 2.5.0 (The PyMOL Molecular Graphics System, version 2.5.0 Schrödinger, LLC, New York, NY, USA).

### 2.4. Use of AI

During the preparation of this work, the authors used ChatGPT version 4.0 to improve the English language of parts of the article. Upon generating draft language, the authors reviewed, edited, and revised the language to their own liking and take ultimate responsibility for the content of this publication. No information or research included in this work was retrieved from AI databases.

## 3. Results and Discussion

### 3.1. Cytotoxicity, Selectivity, and Toxicity of Naphthoquinone Compounds

The cytotoxic potential of eight naphthoquinone compounds was initially evaluated in SCC9 cells, chosen for their high proliferation rate and known phenotype, making them a relevant model for oral squamous cell carcinoma (OSCC). From the initial screening of eight 1,2-naphthoquinone and 1,4-naphthoquinone compounds, only the 1,2-naphthoquinones demonstrated significant cytotoxic effects (Table 2), with compounds **1** (IC_50_ = 2.36 μM), **2** (IC_50_ = 2.026 μM), and **4** (IC_50_ = 1.27 μM) demonstrating IC_50_ values substantially lower than carboplatin (IC_50_ = 181.7 μM) and shikonin (IC_50_ = 3.79 μM). We used carboplatin, an anticancer reference compound used in several different cancer treatments [24,25,26,27], and shikonin, the natural naphthoquinone scaffold from which our benzyl-naphthoquinone derivatives (**1**–**8**) were designed and synthesized, widely recognized for its anticancer activity [5]. The 1,4-naphthoquinones did not reach the threshold for further analysis, as their activity was insufficient to establish reliable dose–response curves; consequently, subsequent experiments focused exclusively on the 1,2-naphthoquinone series. These results demonstrate that the tested 1,2-naphthoquinone compounds are highly effective in reducing cell viability in SCC9 with a clear concentration-dependent effect, justifying further experiments.

To evaluate the impact of tumor heterogeneity on cytotoxic response, all synthesized 1,2-naphthoquinone compounds were assessed in three human oral squamous cell carcinoma (OSCC) cell lines (SCC9, SCC4, and SCC25), as well as in primary human gingival fibroblasts, with the results summarized in Table 3. Across all OSCC models, the compounds reduced cell viability in a dose-dependent manner. The average IC_50_ values were 2.61 µM for SCC-9, 6.37 µM for SCC-4, and 12.18 µM for SCC-25, resulting in an overall mean IC_50_ of 7.06 µM across the three cell lines. These results indicate a strong cytotoxic profile, comparable to or superior to that reported for related quinone-based scaffolds, such as coumarin–naphthoquinone hybrids (average IC_50_ = 56.73 µM), chalcogen-functionalized naphthoquinones (32.28 µM), Mannich adducts of 1,4-naphthoquinones (40.74 µM), and coumarin-1,2,3-triazole conjugates (20.62 µM) [9,23,28].

Among the tumor cell lines, subtle differences in sensitivity were observed, reflecting intrinsic heterogeneity within OSCC subtypes. Compound **1** displayed the lowest IC_50_ value in SCC4 cells (0.95 µM), indicating heightened susceptibility in this model, whereas compound **4** showed the strongest cytotoxic effect in SCC25 cells (IC_50_ = 3.16 µM), suggesting cell-line-dependent variations in drug response. In SCC9 cells, all compounds demonstrated pronounced cytotoxicity, with IC_50_ values in the low micromolar range, reinforcing the robustness of their antitumor activity across distinct OSCC backgrounds.

The selectivity index indicates whether a drug is more toxic to cancer cells or normal cells, thus establishing a safety margin in drug use. The S.I. value for each substance is a ratio obtained with the given formula: S.I. = IC_50_ normal cells/IC_50_ cancer cells. The substances considered selective must present an S.I. ≥ 2, in which lower values of S.I. indicate toxicity towards normal cells [29]. Cytotoxicity evaluation in primary human gingival fibroblasts revealed consistently higher IC_50_ values than those observed in OSCC cell lines for all compounds, indicating a favorable therapeutic window. This selective toxicity toward malignant cells aligns with previous studies demonstrating that naphthoquinone compounds preferentially target cancer cells while sparing non-transformed cells [28,30]. Although the IC_50_ values were in the same micromolar range, compound **1** exhibited the highest average selectivity index across SCC9, SCC4, and SCC25 cells (S.I. = 7.06), markedly higher than those of the reference compounds carboplatin (S.I. = 1.74) and shikonin (S.I. = 1.10), reflecting a pronounced preferential cytotoxicity toward OSCC cells rather than greater intrinsic potency. When compared with other naphthoquinone-based scaffolds previously reported by our and other groups, compound **1** also demonstrated a superior selectivity profile. For instance, acridine-core naphthoquinones presented an overall S.I. of 3.9, chalcogen-functionalized naphthoquinones an S.I. of 3.53, and Mannich adducts derived from 1,4-naphthoquinones an S.I. of 2.35. Collectively, these comparisons highlight the improved tumor selectivity achieved with compound **1** within this chemical class, reinforcing its potential therapeutic relevance [9,23,28]. Carboplatin showed uniformly high IC_50_ values in all OSCC cell lines and fibroblasts, resulting in low selectivity, which is in agreement with its known mechanism of action involving DNA crosslinking and consequent toxicity to both malignant and normal proliferating cells [3]. Similarly, shikonin exhibited potent cytotoxicity in tumor cells but also demonstrated significant toxicity in fibroblasts, yielding low S.I. values and corroborating previous reports that its strong pro-apoptotic effects via oxidative stress and mitochondrial dysfunction limit its clinical applicability due to poor selectivity [31].

Overall, the integrated data presented in Table 3 demonstrate that the synthetic 1,2-naphthoquinones, particularly compound **1**, combine potent cytotoxic activity against multiple OSCC cell lines with a favorable selectivity profile relative to normal fibroblasts. These results are consistent with prior studies on naphthoquinone-based and acridine–naphthoquinone hybrids in SCC models and reinforce the therapeutic relevance of this chemical class as a promising platform for further preclinical anticancer development [22,23]. Although Compound **1** is not novel and has been previously reported in the literature [12], it was originally investigated in the context of neurological models, where it demonstrated relevant in vivo pharmacological activity. However, to the best of our knowledge, it has not been evaluated for anticancer activity, which justifies its investigation in the present study.

**Figure 2:**
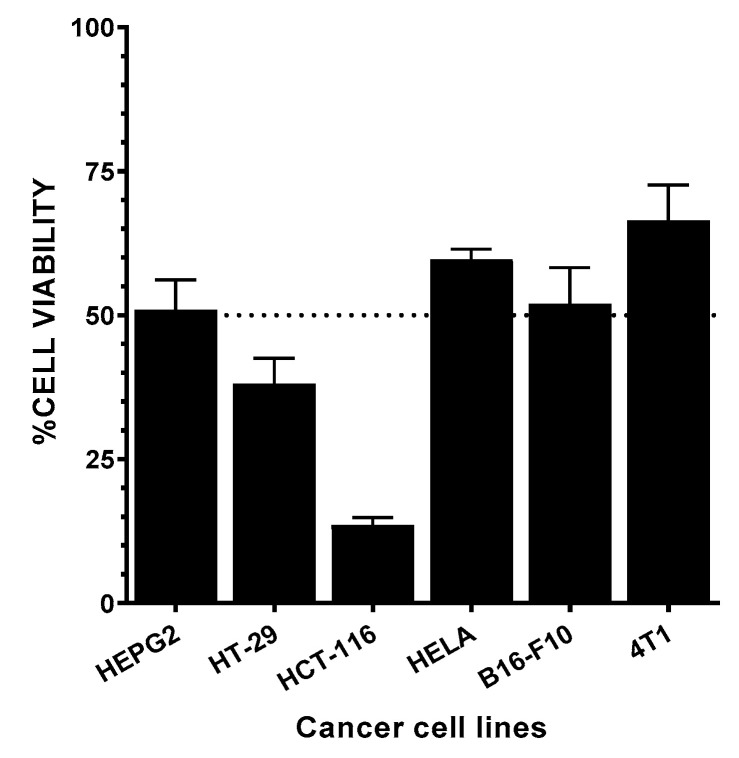
Cytotoxic effects of compound **1** on six different cancer cell lines. Cells (HepG2, HT-29, HCT-116, B16-F10, HeLa, and 4T1) were treated with a concentration corresponding to one-third of the IC_50_ (4.39 µM) determined in primary human fibroblasts. Cell viability was assessed after 48 h of treatment using the MTT assay. The results represent the mean ± standard deviation from three independent experiments.

To further evaluate the selectivity of the most promising compound **1**, its cytotoxic effects were assessed in cancer cell lines from diverse origins, including hepatocarcinoma (HepG2), colon carcinoma (HT-29, HCT-116), cervical carcinoma (HeLa), murine melanoma (B16-F10), and murine breast carcinoma (4T1) (Figure 2). In this experiment, cells were exposed to a concentration equivalent to one-third of the IC_50_ previously determined in primary human fibroblasts (Table 3), allowing evaluation of the compound’s activity across different tumor cell types while minimizing cytotoxic effects on normal cells.

This experiment was designed as a preliminary screening to evaluate whether compound **1** retains cytotoxic activity in additional human cancer cell lines. Although full dose–response curves were not determined for these models, the observed reduction in viability at a concentration corresponding to one-third of the fibroblast IC_50_ indicates that the compound remains active beyond OSCC. The results further showed that compound **1** maintained a selective cytotoxic profile across multiple cancer cell models. Notably, a more pronounced effect was observed in the HCT-116 and HT-29 cell lines, both representative models of human colon adenocarcinoma, suggesting a higher sensitivity of colorectal cancer cells to this compound. These findings support the potential of compound **1** for broader antitumor activity, which should be confirmed in future studies with full dose–response evaluation. This finding is consistent with reports indicating that quinone-based molecules, including 1,2-naphthoquinone compounds, can display tumor-selective cytotoxicity associated with altered redox balance and oxidative stress vulnerability in cancer cells, a feature that is particularly pronounced in colorectal tumors [30]. Similar tumor-selective behavior has been described for other 1,2-naphthoquinone compounds across different cancer cell lines, supporting the relevance of this chemical scaffold as a source of selectively cytotoxic agents with potential applicability to multiple tumor types, including colon cancer [23].

**Figure 3:**
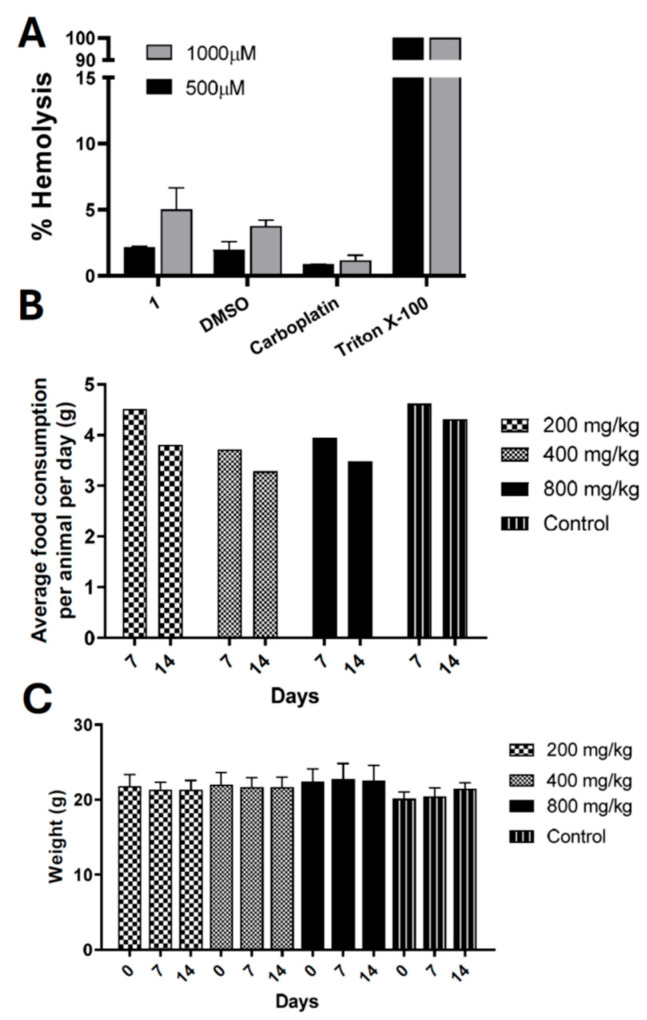
The tolerance of compound **1** was demonstrated both in vitro and in vivo. (**A**) Hemolytic potential of **1**. Graphical representation of the results from treatment of erythrocytes with **1**, DMSO, carboplatin and Triton X-100. (**B**,**C**) Compound **1** exerted low toxicity in C57BL/6 mice. Results from food consumption (**B**) and body weight (**C**) after fourteen days from intraperitoneal administration of compound **1** in different concentrations (200, 400 and 800 mg/kg) and Control (3% DMSO).

To establish more parameters to determine the toxicity of compound **1,** a hemolysis assay was carried out (Figure 3A). It is relevant to define the hemolytic potential because this effect could limit the intravenous administration of drugs in general, limiting their clinical application. The compound induced less than 5% hemolysis at both 500 µM and 1000 µM, indicating negligible erythrocyte toxicity even at high concentrations. Hemolysis levels below 5% are widely regarded as non-toxic in erythrocyte safety assays [32].

The assay controls performed as expected, with the negative control (DMSO) not inducing hemolysis, while the positive control (Triton X-100) caused complete hemolysis, confirming assay reliability and sensitivity. Thus, the maintenance of hemolysis below 5% across all tested concentrations demonstrates the favorable safety profile of compound **1**. These findings are in agreement with previous reports describing minimal hemolytic effects for naphthoquinone compounds [9,19,23]. Therefore, compound **1** emerges as a promising candidate that combines relevant biological activity with a low risk of erythrocyte toxicity.

Beyond the assessment of efficacy and safety, preclinical animal studies are essential for elucidating the in vivo mechanisms of action of newly developed compounds. To determine the tolerated dosage, in animal acute toxicity profile of the most promising molecule, compound **1**, was evaluated in mice using increasing doses of 200, 400, and 800 mg/kg. Macroscopic examinations of the abdominal cavity and major organs during necropsy revealed no observable morphological alterations or lesions in any treated group when compared with control animals. In addition, no significant changes in body weight or food intake were detected at any of the tested doses relative to controls (Figure 3B,C and Table 4).

Consistent with these findings, previous studies have reported similarly low in vivo toxicity for other synthetic naphthoquinone-based compounds [9,19,23]. Although the hemolysis assay and the in vivo acute toxicity evaluation indicated no evident signs of systemic toxicity under the conditions tested, this assessment was limited to acute exposure, macroscopic organ inspection, and monitoring of body weight and food intake. More detailed studies, including histopathological analysis and serum biochemical markers of liver and kidney function, will be necessary to establish a comprehensive safety profile in future investigations. Taken together, the absence of evident dose-limiting toxic and hemolytic effects for compound **1** under the evaluated conditions supports its favorable safety profile and highlights its potential as a candidate for subsequent in vivo anticancer studies at higher dosing regimens, but further confirmations are needed.

The absence of severe adverse effects, even at the highest dose of 800 mg/kg, suggests a wide margin of safety for compound **1**. According to the OECD guidelines for acute oral toxicity, compounds with an LD_50_ above 2000 mg/kg are considered to have low acute toxicity [33]. While higher doses were not evaluated in this study, the current findings indicate that **1** is well tolerated at pharmacologically relevant concentrations. These results further reinforce the favorable safety profile of compound **1**, in line with its low hemolytic activity, supporting its potential for further preclinical development.

### 3.2. Cell Death Pathway Investigation

**Figure 4:**
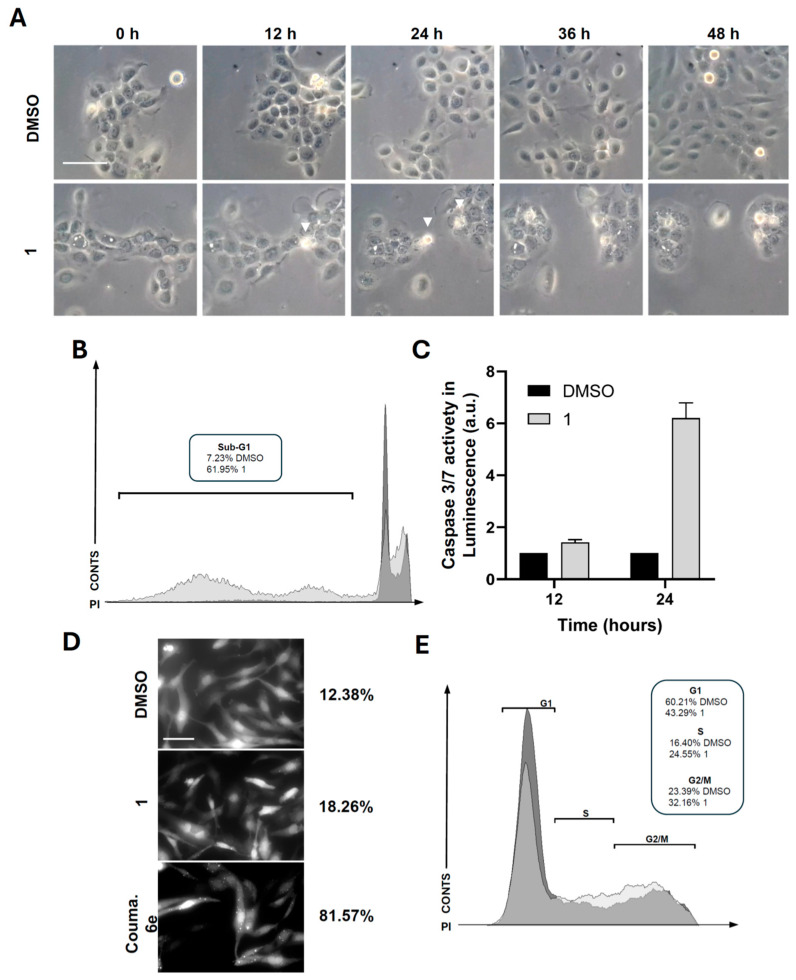
Investigation of cell death–associated events induced by compound **1** in OSCC cells. All experiments were performed using SCC9 cells. (**A**) Time-lapse video microscopy images showing the dynamic behavior of SCC9 cells treated with compound **1** at 2 × IC_50_ (4.72 µM) over a 48 h period, revealing marked morphological alterations and changes in cellular behavior compared with the control group. Images reveal membrane blebs and cell retraction at different times of treatment (white arrows). (**B**) Analysis of DNA fragmentation, indicated by an increased Sub-G1 population, following treatment with compound **1** at 2 × IC_50_ (4.72 µM) after a 48 h incubation. (**C**) Caspase-3/7 activity measured by luminescence in SCC9 cells treated with compound **1** (2 × IC_50_; 4.72 µM), demonstrating a stronger induction of caspase activation at 24 h. (**D**) Evaluation of autophagy induction in SCC9 cells stably expressing GFP-LC3 after treatment with compound **1** for 36 h. Cells treated with DMSO were used as a negative control, while coumarin–naphthoquinone hybrid, Couma.6e, known to induce autophagy in our model [20,34]. Autophagic puncta were visualized by fluorescence microscopy. (**E**) Effect of compound **1** at 2 × IC_50_ (4.72 µM), after 24 h of treatment, on cell cycle distribution in SCC9 cells, highlighting alterations in cell cycle progression. Scale bar is 100 µm for all microscopies. All data are representative of at least three independent experiments.

A key component of drug screening is the identification and characterization of the cell death mechanisms activated by the compound. To investigate the cellular mechanisms underlying the cytotoxic activity of compound **1**, a series of complementary assays was performed in SCC9 cells, integrating live-cell imaging, cell cycle analysis, DNA fragmentation, caspase activation, and autophagy evaluation (Figure 4).

Time-lapse video microscopy was employed to monitor the dynamic response of SCC9 cells treated with compound **1** at 2 × IC_50_ over 48 h. Treated cells progressively lost adhesion, adopted a rounded morphology, and exhibited a gradual reduction in cytoplasmic volume, followed by late-stage structural fragmentation and membrane irregularities compatible with apoptotic-like body formation. In contrast, DMSO-treated cells preserved stable morphology and adhesion throughout the observation period (Figure 4A).

The temporal sequence of cell shrinkage, rounding, and late-stage fragmentation observed here corresponds to classical apoptotic dynamics described in live-cell imaging studies. Similar morphological trajectories have been documented using advanced time-lapse and digital detection approaches, supporting the interpretation that compound **1** induces an apoptosis-related process rather than nonspecific necrotic damage [34].

To further characterize the mode of cell death, DNA fragmentation was assessed by quantifying the Sub-G1 population following treatment with compound **1** at 2 × IC_50_. A pronounced increase in the Sub-G1 fraction was observed in treated cells (61.95%) compared with the DMSO control (7.23%), indicating extensive DNA fragmentation (Figure 4B). The substantial accumulation of Sub-G1 cells provides strong evidence for apoptotic DNA degradation, a hallmark of programmed cell death. This finding complements the morphological alterations detected by time-lapse microscopy and aligns with previous reports describing apoptosis induction by naphthoquinone compounds in cancer cells [23].

To directly assess apoptotic signaling, caspase-3/7 activity was quantified by luminescence after 12 and 24 h of exposure to compound **1**. A moderate increase in caspase activity was detected at 12 h, while a markedly stronger induction was observed at 24 h compared with the DMSO control (Figure 4C). The time-dependent activation of executioner caspases indicates sustained engagement of apoptotic pathways rather than a transient stress response. The increase in Sub-G1 population, activation of caspase-3/7, and morphological changes observed by time-lapse microscopy are consistent with apoptosis as a major mechanism of cell death under the conditions tested. However, these experiments were not designed to distinguish between intrinsic and extrinsic apoptotic pathways or to fully exclude the contribution of other regulated cell death mechanisms. Further studies employing pathway-specific assays will be required to elucidate these aspects in detail. Similar delayed yet robust caspase-3/7 activation profiles have been reported for other naphthoquinone compounds, including β-lapachone and shikonin, which induce apoptosis [22,23,35]. Autophagy is a well-recognized cellular stress response and an important form of regulated cell death, particularly in the context of anticancer therapies. Therefore, assessing autophagic activity was necessary to determine whether this pathway contributes to the cytotoxic effects of compound **1** or could be ruled out as a dominant mechanism. Autophagy was evaluated by GFP–LC3 puncta formation as a screening approach. Additional treatment times (6, 12, 24, and 48 h) were also tested, and no significant increase in detectable GFP–LC3 puncta was observed in cells treated with compound **1** at any of these time points. The 36 h time point was selected for presentation because the positive control displayed the most pronounced staining under these conditions.

Under the conditions tested, compound **1** did not significantly increase puncta formation compared with the control, suggesting that strong activation of autophagy is unlikely in this model. However, this assay is semi-quantitative and does not directly measure autophagic flux; therefore, the involvement of autophagy cannot be completely excluded. Further studies using flux markers such as LC3-II and p62 will be necessary to confirm these findings.

Semi-quantitative analysis showed that LC3-positive puncta were detected in 18.26% of treated cells, a value comparable to the DMSO control (12.38%). In contrast, the positive control, the coumarin–naphthoquinone hybrid Couma.6e [20], induced a strong autophagic-like response in 81.57% of cells (Figure 4D). The absence of a meaningful increase in LC3 puncta formation indicates that compound **1** does not significantly activate autophagy under the tested conditions. This result suggests that autophagy is not a major contributor to cell death in this model and is consistent with previous studies showing that naphthoquinone compounds preferentially trigger apoptosis rather than autophagic cell death, often through ROS-mediated and mitochondria-dependent mechanisms [36,37].

Cell cycle analysis was conducted to evaluate whether compound **1** affects cell cycle progression and cellular proliferation. Treatment with compound **1** led to a marked reduction in the G1 population (from 60.16% to 42.27%), together with an accumulation of cells in the S phase (from 16.40% to 22.55%) and in the G2/M phase (from 23.39% to 30.16%) when compared with the DMSO control (Figure 4E). These shifts indicate that compound **1** interferes with normal cell cycle progression, promoting arrest at the S and G2/M phases and thereby impairing DNA replication and/or mitotic entry. This cell cycle disruption is consistent with observations from the time-lapse assay, in which control cells displayed a pronounced increase in cell number over time, reflecting active proliferation, whereas compound **1**–treated cells failed to expand and progressively decreased in number. Together, these findings suggest that compound **1** suppresses cell proliferation through cell cycle arrest, an effect that likely contributes to the subsequent activation of apoptotic pathways. Such coupling between cell cycle blockade and apoptosis is a well-established mechanism for quinone-based compounds [38,39].

### 3.3. Predicted Pharmacokinetic and Physicochemical Characteristics of Compound ***1***

Computational evaluation of pharmacokinetic and physicochemical properties based on molecular structure represents an essential step in predicting how candidate compounds may behave in biological systems. Such in silico analyses aid in prioritizing promising molecules, contributing to the rational design of safer drugs and helping to minimize the risk of adverse outcomes in subsequent in vivo studies [40]. Widely used platforms for this purpose include SwissADME and admetSAR, which offer valuable predictions related to absorption, distribution, and oral bioavailability.

According to Lipinski’s Rule of Five, four key parameters are considered when evaluating the suitability of a compound for oral administration (Table 5): (1) the n-octanol/water partition coefficient (cLogP ≤ 5); (2) the number of hydrogen bond acceptors (nON ≤ 10); (3) the number of hydrogen bond donors (nOH/NH ≤ 5); and (4) molecular weight (MW ≤ 500 Da). The overall drug-likeness is reflected by the number of violations, as compounds presenting two or more deviations are typically considered unfavorable for oral delivery. In this evaluation, compound **1** presented no violations, indicating a favorable predicted profile for oral absorption, consistent with reports for other previously studied naphthoquinone derivatives [9,23]. In contrast, shikonin exhibited three violations, whereas carboplatin complied with all four criteria without any deviations.

Another key descriptor for predicting membrane permeability and oral absorption is the topological polar surface area (TPSA). Because biological membranes are predominantly lipidic, effective drug candidates must maintain a balance between polarity and lipophilicity to enable passive diffusion across lipid bilayers. Compounds with TPSA values exceeding 140 Å^2^ are generally associated with poor membrane permeability, whereas those with TPSA values below 60 Å^2^ tend to exhibit more efficient transmembrane diffusion [41].

Within this framework, compound **1**, with a TPSA of 46.7 Å^2^, falls within the range considered favorable for passive membrane permeation. Combined with its moderate lipophilicity (cLogP = 2.58), this profile suggests a balanced relationship between polarity and lipophilicity, two physicochemical features often associated with efficient membrane permeability and potential oral absorption [42]. In comparison, carboplatin (TPSA = 126.64 Å^2^; cLogP = −1.79) and shikonin (TPSA = 94.83 Å^2^; cLogP = 2.05) present physicochemical characteristics that are generally less favorable for passive diffusion, particularly in the case of carboplatin, whose higher polarity may limit membrane permeability. These differences highlight the comparatively favorable permeability-related profile predicted for compound **1** [43].

To further explore the pharmacokinetic profile, additional ADMET parameters were predicted using the admetSAR platform (Table 6). Compound **1** was predicted to present human intestinal absorption and Caco-2 permeability, suggesting potential compatibility with oral exposure. The compound was also predicted to act as a P-glycoprotein inhibitor but not as a P-glycoprotein substrate, indicating a possible reduced susceptibility to efflux-mediated elimination compared with compounds that are substrates of this transporter. In contrast, carboplatin was predicted to have limited Caco-2 permeability and no interaction with P-glycoprotein as inhibitor or substrate, whereas shikonin showed predicted intestinal absorption and Caco-2 permeability but no P-glycoprotein inhibition.

Predictions of mean residence time (MRT) did not indicate a strong tendency toward prolonged systemic persistence for compound **1**, and these values should be interpreted cautiously, as they are model-based estimates. Collectively, these physicochemical and predicted ADMET properties suggest that compound **1** may be compatible with oral absorption and systemic exposure relative to the reference compounds; however, these predictions are based on in silico descriptors and should be confirmed by experimental pharmacokinetic studies [43].

Taken together, the in silico analyses indicate that compound **1** integrates physicochemical and absorption-related features that are consistent with those generally associated with compactible orally active small molecules. The observed balance between polarity and lipophilicity suggests favorable membrane interaction and pharmacokinetic behavior, as supported by the predicted intestinal absorption and Caco-2 permeability. However, as these parameters are derived from computational models, experimental pharmacokinetic studies will be necessary to confirm these properties. Overall, these attributes support the continued pharmacological and preclinical evaluation of compound **1** [44].

### 3.4. Analysis of Putative Antitumor Targets of Compound ***1*** by In Silico Reverse Docking

**Figure 5:**
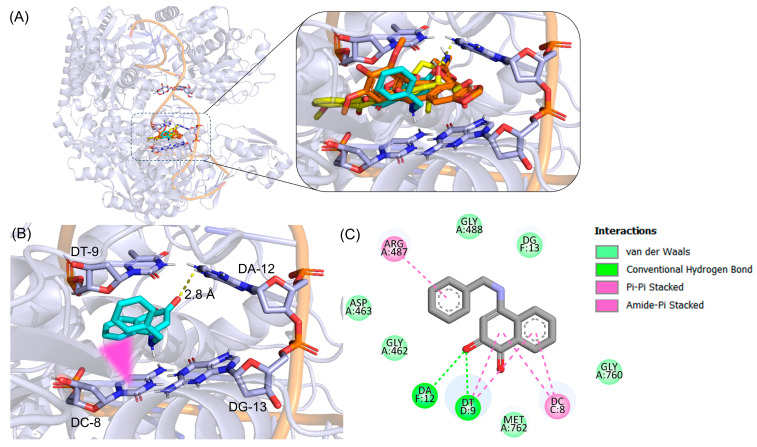
Binding mode and interactions of compound **1** in comparison to known ligands of topoisomerase IIα. (**A**) Superposition of the drug doxorubicin (in yellow), the co-crystallized etoposide (in orange), and compound **1** (in cyan) in the active site of topoisomerase IIα-DNA (in white). Yellow dashes in the image represent hydrogen bonding interactions (**B**) Main interactions of compound **1** with the residues of active site. Yellow dashes in the image represent hydrogen bonds and pink triangles represent π–π stacking interactions. (**C**) Interaction map of compound **1** in 2D format carried out in the Discovery Studio Visualizer v24.1.0.23298 program.

In an attempt to find potential targets for the naphthoquinone compounds studied here, we conducted a molecular docking study considering protein targets of known naphthoquinones, which are related to cancer. Some of these targets have already been studied by our group previously [9,19,20,21,22,23,45]. The three-dimensional structures of these proteins were found in the RCSB Protein Data Bank under the following codes: topoisomerase IIα ATPase domain (PDB 1ZXM), ribosomal protein S6 kinase 2 (RSK2; PDB 4NW6), topoisomerase I DNA-binding domain (PDB 1K4T), topoisomerase IIα (PDB 5GWK), and topoisomerase IIβ (PDB 3QX3). Human pyruvate kinase M2 (Uniprot P14618) in the closed conformation was constructed by homology modeling using the Swiss-model online server, and the pyruvate kinase M2 from *Oryctolagus cuniculus* (PDB 1A49) co-crystallized with ATP was chosen as a template. The interaction between compound **1** and the ATPase domain of topoisomerase IIα was also analyzed and compared with the binding mode of 1,4-naphthoquinone and the co-crystallized ligand AMP-PNP, a non-hydrolyzable ATP analog. When compared with the co-crystallized ligand, compound **1** overlapped, but we did not observe the same hydrogen bond described by Wei et al. (2005) as important and responsible to stabilize the 2′-hydroxyl group to the S149 side chain [46]. However, the hydrogen bond that stabilizes the 3′-hydroxyl to the N150 side chain was observed. When compared with 1,4-naphthoquinone, compound **1** did not overlap. Compound **1** presents a higher binding energy (−9.0 kcal mol^−1^) than that of the co-crystallized ligand (−11.8 kcal mol^−1^), indicating lower affinity. 1,4-naphthoquinone exhibits an even higher binding energy value (−7.3 kcal mol^−1^), probably due to its smaller size and consequent fewer interactions with the active site. Thus, due to its different binding mode and its lower interaction affinity when compared to the co-crystallized ligand, it is believed that **1** does not have its effect through this molecular target. Compound **1** was evaluated with the DNA-binding domain of topoisomerases I, IIα, and IIβ. For comparison purposes, doxorubicin, an anticancer drug used in the treatment of various types of cancer, which targets topoisomerases I, IIα, and IIβ was evaluated in the same computational protocol. The co-crystallized ligand and compound **1** overlapped in the DNA-binding domain of topoisomerase I, but **1** showed higher binding energy (−9.5 kcal mol^−1^) when compared to redocking of the co-crystallized ligand (−11.3 kcal mol^−1^) and doxorubicin docking (−10.0 kcal mol^−1^). Doxorubicin is known to inhibit DNA topoisomerases through intercalation with DNA, as well as some quinones [47,48,49], with base pair interactions being of fundamental importance for target inhibition. Therefore, it is considered that the compound in question does not target DNA, since it does not establish base pair interactions, which are essential for target inhibition. In topoisomerase IIα, the docking of **1** showed that it overlapped with the drug doxorubicin and the ligand etoposide. Although they were not fully aligned, doxorubicin and compound **1** paired with the nitrogenous bases DC8-DG13 and DT9-DA12, the latter through a hydrogen bond with the naphthoquinone moiety of **1**. Furthermore, the π–π stacking interaction between DT9 and DC8 with the naphthoquinone rings reinforces the importance of this interaction in the intercalation process (Figure 5). When compared with the co-crystallized ligand etoposide, **1** overlapped and maintained the same contacts with the nitrogenous bases, but the studied compound maintained these contacts through hydrogen bonds, suggesting a stronger interaction. It is known that etoposide’s inhibition of topoisomerases II occurs through the interaction of the dioxolane and benzene rings of the compound with the enzyme, in addition to the interaction between 2,6-dimethoxyphenol ring and the enzyme [50]. It is precisely at these points that the studied compound **1** binds to the topoisomerase II in a similar manner to the drug etoposide, with the naphthoquinone moiety overlapping the 2H-1,3-benzodioxole rings and the benzene of **1** overlapping the 2,6-Dimethoxyphenol ring of etoposide (Supplementary Material Figure S27). Although compound **1** exhibits higher binding energy (−9.0 kcal mol^−1^) when compared to the drug doxorubicin (−10.2 kcal mol^−1^) and the redocking of the co-crystallized ligand (−10.3 kcal mol^−1^), the values were close, and the interactions observed between the ligands and the nitrogenous bases and their insertion into the active site suggests the DNA-binding domain of topoisomerase IIα seems to be a target for compound **1**.

**Figure 6:**
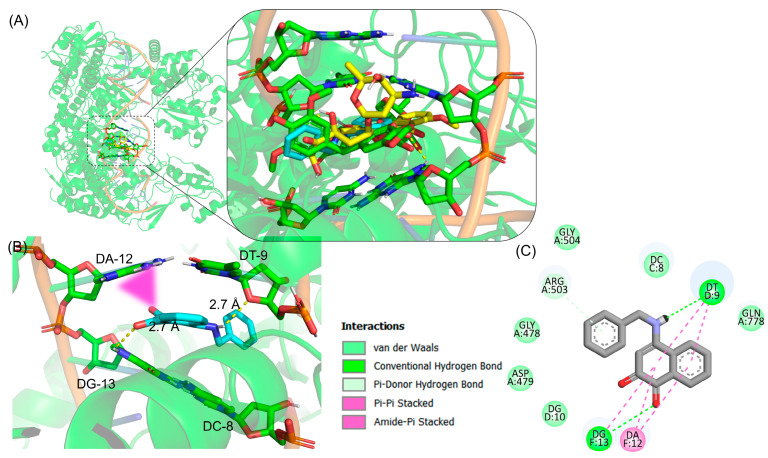
Binding mode and interactions of compound **1** in comparison to known ligands of topoisomerase IIβ. (**A**) Superposition of the drug doxorubicin (in yellow), the co-crystallized etoposide (in green), and compound **1** (in cyan) in the active site of topoisomerase IIβ-DNA. Yellow dashes in the image represent hydrogen bonding interactions. (**B**) Main interactions of compound **1** with the residues of active site. Yellow dashes in the image represent hydrogen bonding interactions and pink triangles represent π–π stacking interactions. (**C**) Interaction map of compound **1** in 2D format carried out in the Discovery Studio Visualizer v24.1.0.23298 program.

Molecular modeling of **1** was also carried out in the DNA binding domain of topoisomerase IIβ and the co-crystallized ligand etoposide and the drug doxorubicin were tested for comparison. The molecular docking results of compound **1** in comparison to the co-crystallized ligand showed that the aromatic moiety is aligned with the 2,6-dimethoxyphenol group of etoposide, which is one of the main groups responsible for the interaction with topoisomerase (Supplementary Material Figure S28) [51]. When compared to doxorubicin, we found the naphthoquinone moiety of **1** aligned with the polyaromatic system of anthracycline present in doxorubicin, which is responsible for the intercalation with DNA [51]. Compound **1** interacts through the naphthoquinone moiety with the nitrogenous bases DT9 through hydrogen bonding interaction and through π–π stacking with DA12. (Figure 6). We also found a hydrogen bond interaction between the carbonyl present in the naphthoquinone moiety and compound **1**, and van der Waals interactions between the nitrogenous base DC8. Although compound **1** exhibits a higher binding energy (−9.9 kcal mol^−1^) than the reference drug doxorubicin (−11.5 kcal mol^−1^) and the co-crystallized ligand (−14.6 kcal mol^−1^), it is believed that it can interact with this target, since it maintains similar interactions. Furthermore, the smaller size of the studied structure may directly affect its interaction energy, as it contains fewer atoms and therefore may have a smaller contact area.

**Figure 7:**
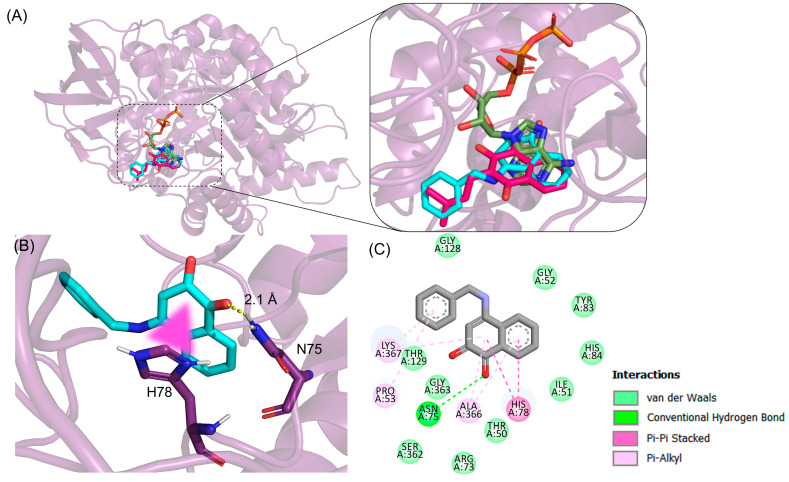
Binding mode and interactions of compound **1** in comparison to known ligands of PKM2. (**A**) Superposition of the lapachol (in pink) and ATP (in green) and compound **1** (in cyan) in the active site of PKM2. (**B**) Main interactions of compound **1** with the residues of active site. Yellow dashes in the image represent hydrogen bonds and pink triangles represent π–π stacking interactions. (**C**) Interaction map of compound **1** in 2D format carried out in the Discovery Studio Visualizer v24.1.0.23298 program.

PKM2 is an interesting target for antineoplastic treatments due to its crucial role as a metabolic regulator in tumor cells, as well as being positively regulated by cancer cells [52]. Shikonin [53,54,55,56], lapachol [57], and the co-crystallized ATP [58], which have already demonstrated inhibitory activity in protein kinase PKM2, were subjected to the same computational protocol for comparison. Compound **1** exhibited a higher binding energy (−8.5 kcal mol^−1^) when compared to ATP (−9.4 kcal mol^−1^, but it presented a lower binding energy when compared to the known inhibitors lapachol (−7.7 kcal mol^−1^) and shikonin (−8.0 kcal mol^−1^). The comparison of compound **1** with ATP and lapachol in the binding site revealed that the naphthoquinone moiety occupied the same site as the aromatic nucleus of ATP, maintaining contact with residue N75 by hydrogen bond, as ATP does, while lapachol contacts the same residue by van der Waals interaction (Supplementary Material Figure S29). The naphthoquinone moiety of lapachol, ATP, and compound **1** made π–π stacking interaction with residue H78, which seems to be important for the enzyme inhibition (Figure 7).

Thus, considering the ATP-like binding mode in the PKM2-ATP complex and the similar interactions with the target, comparing the studied compound to ATP and lapachol, the interactions predicted by the molecular docking simulations suggest that compound **1** may interact with the target in a similar way to the other ligands already studied.

**Figure 8:**
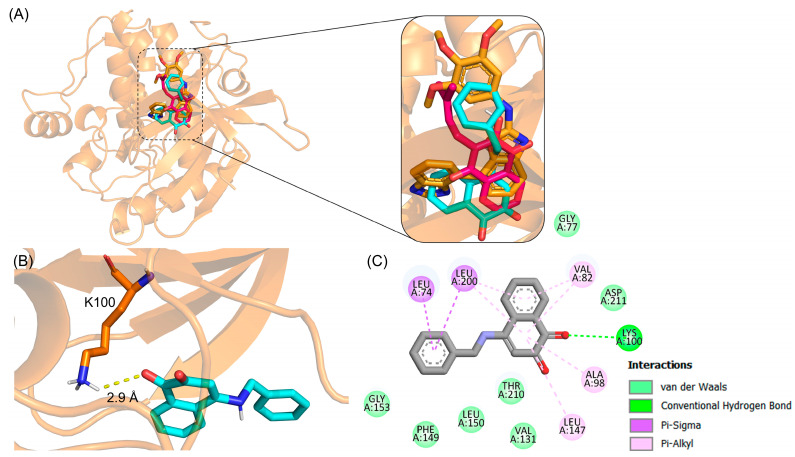
Binding mode and interactions of compound **1** in comparison to known ligands of RSK2. (**A**) Superposition of the lapachol (in pink), the co-crystallized 7-(1H-benzimidazol-7-yl)-N-(3,4,5-trimethoxyphenyl)-1,3-benzoxazol-2-amine (in gold), and compound **1** (in cyan) in the active site of topoisomerase IIα-DNA. (**B**) Main interactions of compound **1** with the residues of the active site. Yellow dashes in the image represent hydrogen bonds. (**C**) Interaction map of compound **1** in 2D format carried out in the Discovery Studio Visualizer v24.1.0.23298 program.

Ribosomal protein S6 kinase 2 (RSK2) is responsible for regulating several cellular processes, such as proliferation, cell cycle progression, and apoptosis. For comparative purposes, we used lapachol [59] in the same computational protocol. This compound triggers cytotoxicity in squamous cell carcinoma by inhibiting RSK2. The docking results of **1** on RSK2 were compared with the co-crystallized ligand, 7-(1H-benzimidazol-7-yl)-N-(3,4,5-trimethoxyphenyl)-1,3-benzoxazol-2-amine, and with lapachol (Supplementary Material Figure S30). We observed that **1** binds to RSK2 in a similar manner to lapachol, but not identically, since it did not maintain the same interaction pattern, presenting a hydrogen bond interaction with K100 (Figure 8). Aronchik et al. (2014) showed interaction with the catalytic K100 residue of the protein as important for the inhibition [60]. In kinases, this K100 residue in the catalytic domain is often highly conserved and essential for enzymatic activity because it helps position ATP and stabilize negative charges during phosphate transfer [61]. We found lapachol and the co-crystallized ligand interacting by a hydrogen bond with L150, which appears to be important for the binding of several RSK2 inhibitors [60,62], while **1** showed a van der Waals interaction with the same residue (Figure 8).

Although **1** presents a lower binding energy (−9.0 kcal mol^−1^) compared to lapachol (−8.1 kcal mol^−1^), it has a higher binding energy when compared to the redocking of the co-crystallized ligand (−10.3 kcal mol^−1^), which can be justified by its smaller size when compared to the co-crystallized ligand 7-(1H-benzimidazol-7-yl)-N-(3,4,5-trimethoxyphenyl)-1,3-benzoxazol-2-amine. Thus, through the observed data, this result indicates that RSK2 may be the molecular target of the compound, since the observed interaction pattern is compatible with the inhibition mode described for this protein and performs a key interaction in the catalytic site.

Herein, molecular docking was used as a tool for identifying potential targets for compound **1** and exploring possible interactions. The present study suggests that com-pound **1** presents a similar binding mode to previously described naphthoquinones with the DNA domain of topoisomerase IIα and IIβ, RSK2, and PKM2. Thus, molecular docking data should be interpreted as initial evidence of the possibility of structural interaction, contributing to the search for hypothetical potential targets. Confirmation of these results may be done by future experimental studies.

## 4. Conclusions

A series of eight 1,2-naphthoquinone and 1,4-naphthoquinone derivatives were synthesized and screened for anticancer activity; only the 1,2-naphthoquinones demonstrated significant cytotoxic effects, leading to the identification of compound **1** as the most promising candidate based on its potent and selective cytotoxicity toward OSCC cells and other types of cancer. Compound **1** displayed an apparent favorable safety profile, with negligible hemolytic activity and low acute toxicity in vivo, showing no mortality or severe adverse effects in mice at doses up to 800 mg/kg. Although the results indicate that compound **1** presents low acute toxicity under the conditions evaluated, further studies, including histopathological and biochemical analyses, are required to fully characterize its safety profile.

Mechanistically, compound **1** induced apoptosis-like cell death, as evidenced by time-dependent caspase-3/7 activation, accumulation of cells in the S and G2/M phases, and a marked increase in the Sub-G1 population. The available data do not indicate a significant activation of autophagy under the tested conditions, however, additional experiments assessing autophagic flux will be required to definitively determine the role of this pathway. This indicates that apoptosis is the predominant mode of cell death.

In silico pharmacokinetic analyses further highlighted the drug-like nature of compound **1**, which complied fully with Lipinski’s Rule of Five and exhibited favorable physicochemical properties, including optimal TPSA and moderate lipophilicity, predictive of good membrane permeability and oral bioavailability. Reverse docking studies suggested that its antitumor activity may involve multiple cancer-relevant targets, including topoisomerase IIα/β, PKM2, and RSK2, providing a plausible molecular basis for its effects on cell cycle progression, metabolic regulation, and apoptotic signaling.

Collectively, these findings demonstrate that compound **1** integrates potent and selective anticancer activity with a well-defined apoptosis-driven mechanism of action, favorable pharmacokinetic predictions, and a reassuring safety profile both in vitro and in vivo. This combination of properties positions compound **1** as a compelling lead candidate for further preclinical evaluation and optimization, with strong potential for development as a novel chemotherapeutic agent for OSCC and possibly other malignancies.

## Figures and Tables

**Scheme 1:**
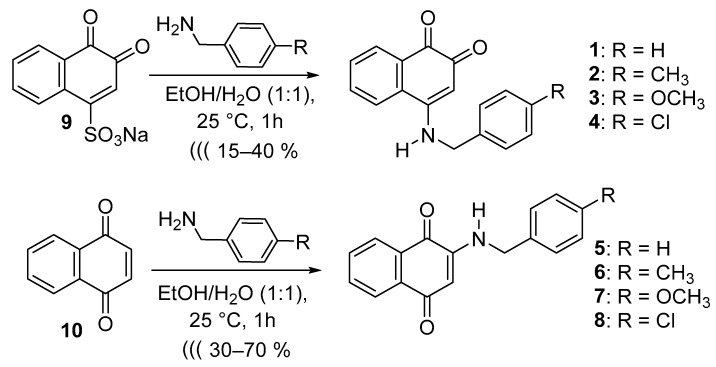
Synthetic route used to prepare compounds **1**–**8**.

**Table 1:** Information about molecular docking protocol of each protein.

Target	RSK2	TopoIDNA	TopoIIaATPase	TopoIIaDNA	TopoIIbDNA	PKM2
PDB	4NW6	1K4T	1ZXM	5GWK	3QX3	1A49 X 3SRD
Co-crystallized ligand	2-amino-7-substituted benzoxazole	Topotecan	AMP-PNP	Etoposide	Etoposide	ATP
Exhaustiveness	8	8	8	8	8	5
Dimensions (Å)	24 × 18 × 20 Å	20 Å^3^	26 Å^3^	18 Å^3^	20 Å^3^	18 × 24 × 20 Å
Center (Å)	−17.217 × −0.699 × −19.500 Å	21.541 × −3.558 × 27.791 Å	35.933 × 0.377 × 36.838 Å	23.7 × −38.695 × −60.34 Å	32.884 × 95.413 × 50.785 Å	−9.975 × 57.403 × 11.74 Å
Redocking (kcal/mol^−1^)	−10.3	−11.3	−11.8	−10.3	−14.6	−9.4
RMSD (Å)	0.495	0.271	0.213	0.253	0.103	1.685

**Table 2:** IC_50_ determination of the eight synthetic 1,2 and 1,4-naphthoquinone compounds (**1**–**8 compounds**). The OSCC cell line, SCC9, was treated with the indicated compounds for 48 h and cell viability was determined by the MTT assay. Shown from left to right: compound nomenclature, IC_50_ (μM), and Standard Deviation (SD) from at least 3 independent experiments. N.D. stands for not determined at the tested concentration.

Compounds	IC_50_	SD
**1**	2.36	0.04
**2**	2.02	0.05
**3**	4.81	0.14
**4**	1.27	0.08
**5**	ND	ND
**6**	ND	ND
**7**	ND	ND
**8**	ND	ND
**Shikonin**	3.79	0.04
**Carboplatin**	181.7	0.03

**Table 3:** 1,2-naphthoquinone compounds are selectively cytotoxic to three different oral cancer cell lines. Half-maximal inhibitory concentration (IC_50_) in other human OSCC is indicated. Human gingival fibroblasts were used to calculate the selectivity index (SI). SD = standard deviation. Statistical data are representative of three different experiments. Comparisons discussed in the text refer to differences in selectivity index (SI) rather than direct statistical comparisons of IC_50_ values.

Compounds		Cancer OSCC Cells								Primary Gingival Fibroblast		Average S.I.
	SCC9			SCC4			SCC25					
	IC_50_ (µM)	S.D.	S.I.	IC_50_ (µM)	S.D.	S.I.	IC_50_ (µM)	S.D.	S.I.	IC_50_ (µM)	S.D.	
**1**	2.36	0.04	5.58	0.95	0.09	13.86	7.21	0.07	1.83	13.17	0.11	7.06
**2**	2.02	0.05	10.8	10.82	0.1	2.01	13.07	0.1	1.67	21.8	0.10	4.82
**3**	4.81	0.14	4.36	3.13	0.05	6.7	25.29	0.07	0.83	21.0	0.09	3.96
**4**	1.27	0.08	5.70	10.57	0.11	0.68	3.16	0.13	2.29	7.25	0.14	2.88
**Shikonin**	3.79	0.04	0.27	0.83	1.41	1.26	0.58	0.05	1.79	1.04	0.03	1.10
**Carboplatin**	181.7	0.03	1.41	141.5	0.2	1.81	128.5	0.03	2.00	256.9	0.04	1.74

**Table 4:** Acute toxicity study: morbidity, mortality, gross organs necropsy, and histology of mice treated with compound **1**.

Treatment	Dose mg/kg ^a^	Change in Body Weight	Morbidity ^b^	Mortality	Gross Necropsy ^c^
**Control**	0 N = 3	Absent	Normal	Absent	No alteration
**1**	200 N = 3	Absent	Normal	Absent	No alteration
	400 N = 3	Absent	Normal	Absent	Adhesion of abdominal cavity organs
	800 N = 3	Absent	Normal	Absent	Adhesion of abdominal cavity organs

^a^ Dose level for each subsequent group was determined based on the result after the previous lower dosing. We started at 200 mg/kg and proceeded to double to 400 and 800 mg/kg. N is the number of animals used for each experiment. ^b^ Animals were monitored every day, twice a day for observation of morbidity symptoms that were considered: posture, eye appearance, tremors, convulsion, diarrhea, response to external stimuli, signs of pain, and mobility defect. ^c^ Gross organ necropsy of the liver, thymus, right kidney, right testicle, major lymph nodes, lungs, and heart.

**Table 5:** Predicted safety profile and SwissADME-derived physicochemical parameters of **1** in comparison with doxorubicin and carboplatin. Properties related to Lipinski’s “Rule of Five” (CLogP, nON, nOH/NH and MW), is shown. Compliance with each rule is indicated as Accepted or Non-accepted.

Compounds	Physicochemical Properties					
	CLogP	nON	nOH/NH	MW	Lipinski’s Rule Violations ^a^	TPSA (Å^2^)
**1**	2.58	2	1	263.3	Accept	46.7
**Shikonin**	2.05	5	3	288.3	Accept	94.83
**Carboplatin**	−1.79	6	4	371.2	Accept	126.64

^a^ Violations to the Lipinski “rule of 5”: CLogP ≤ 5; MW ≤ 500; nON, number of hydrogen bond acceptors ≤ 10; and nOH/NH, number of hydrogen bond donors ≤ 5.

**Table 6:** Properties analyzed in admetSAR. The properties analyzed were oral bioavailability, substrate, and P-gp inhibitor. The analysis was performed with compound **6a** and the chemotherapeutic agents carboplatin and doxorubicin as controls.

ADMET	Compound 1	Carboplatin	Shikonin
Human intestinal absorption	1	1	1
P-glycoprotein inhibitor	1	0	0
P-glycoprotein substrate	0	0	0
Caco-2 permeability	1	0	1
Mean Residence Time	−0.89	−0.69	0.05

## Data Availability

Data will be available upon request.

## Supplementary Materials

The following supporting information can be downloaded at: https://www.mdpi.com/article/10.3390/biomedicines14040757/s1, Figure S1. IR of 4-(benzylamino)naphthalene-1,2-dione (1). Figure S2. ^1^H NMR (500 MHz. DMSO-*d_6_*) of 4-(benzylamino)naphthalene-1,2-dione (1). Figure S3. HRMS of 4-(benzylamino)naphthalene-1,2-dione (1). Figure S4. IR of 4-((4-methylbenzyl)amino)naphthalene-1,2-dione (2). Figure S5. ^1^H NMR (500 MHz. DMSO-*d_6_*) of 4-((4-methylbenzyl)amino)naphthalene-1,2-dione (2). Figure S6. ^13^C NMR/APT (125 MHz. DMSO-*d_6_*) of 4-((4-methylbenzyl)amino)naphthalene-1,2-dione (2). Figure S7. HRMS of 4-((4-methylbenzyl)amino)naphthalene-1,2-dione (2). Figure S8. IR of 4-((4-methoxybenzyl)amino)naphthalene-1,2-dione (3). Figure S9. ^1^H NMR (500 MHz. MeOD) of 4-((4-methoxybenzyl)amino)naphthalene-1,2-dione (3). Figure S10. HRMS of 4-((4-methoxybenzyl)amino)naphthalene-1,2-dione (3). Figure S11. IR of 4-((4-chlorobenzyl)amino)naphthalene-1,2-dione (4). Figure S12. ^1^H NMR (500 MHz. DMSO-*d_6_*) of 4-((4-chlorobenzyl)amino)naphthalene-1,2-dione (4). Figure S13. HRMS of 4-((4-chlorobenzyl)amino)naphthalene-1,2-dione (4). Figure S14. IR of 2-(benzylamino)naphthalene-1,4-dione (5). Figure S15. ^1^H NMR (500 MHz. CDCl_3_) of 2-(benzylamino)naphthalene-1,4-dione (5). Figure S16. HRMS of 2-(benzylamino)naphthalene-1,4-dione (5). Figure S17. IR of 2-((4-methylbenzyl)amino)naphthalene-1,4-dione (6). Figure S18. ^1^H NMR (500 MHz. CDCl_3_) of 2-((4-methylbenzyl)amino)naphthalene-1,4-dione (6). Figure S19. HRMS of 2-((4-methylbenzyl)amino)naphthalene-1,4-dione (6). Figure S20. IR of 2-((4-methoxybenzyl)amino)naphthalene-1,4-dione (7). Figure S21. ^1^H NMR (500 MHz. CDCl_3_) of 2-((4-methoxybenzyl)amino)naphthalene-1,4-dione (7). Figure S22. HRMS of 2-((4-methoxybenzyl)amino)naphthalene-1,4-dione (7). Figure S23. IR of 2-((4-chlorobenzyl)amino)naphthalene-1,4-dione (8). Figure S24. ^1^H NMR (500 MHz. CDCl_3_) of 2-((4-chlorobenzyl)amino)naphthalene-1,4-dione (8). Figure S25. HRMS of 2-((4-chlorobenzyl)amino)naphthalene-1,4-dione (8). Figure S26. Chemical structure of Couma.6e, a previously described naphthoquinone–triazole–coumarin hybrid used in this study as a positive control for autophagy induction. Chemical name: 2-methyl-3-((4-(((2-oxo-2H-chromen-4-yl)oxy)methyl)-1H-1,2,3-triazol-1-yl)(phenyl)methyl)-naphthalene-1,4-dione (Couma. 6e). Figure S27. Molecular docking of the compound 1, the drug doxorubicin and the co-crystallized ligand etoposide in the active site of topoisomerase IIα-DNA. Compound 1 is represented in cyan, drug doxorubicin is represented in yellow, the co-crystallized ligand etoposide is represented in orange. Yellow dashes in the image represent hydrogen binding interactions and pink triangles represent ππ-stacking interactions. Figure S28. Molecular docking of the compound 1, the drug doxorubicin and the co-crystallized ligand etoposide in the active site of topoisomerase IIβ-DNA. Compound 1 is represented in cyan, drug doxorubicin is represented in yellow, the co-crystallized ligand etoposide is represented in green. Yellow dashes in the image represent hydrogen binding interactions and pink triangles represent ππ-stacking interactions. Figure S29. Molecular docking of the compound 1, the inhibitor lapachol and ATP in the active site of PKM2. Compound 1 is represented in cyan, lapachol is represented in pink, and ATP is represented in green. Yellow dashes in the image represent hydrogen binding interactions and pink triangles represent π-π-stacking interactions. Figure S30. Molecular docking of the compound 1 (E = −9.0 kcal mol^−1^), the inhibitor lapachol and 7-(1H-benzimidazol-7-yl)-N-(3,4,5-trimethoxyphenyl)-1,3-benzoxazol-2-amine in the active site of RSK2. Compound 1 is represented in cyan, lapachol is represented in pink, and 7-(1H-benzimidazol-7-yl)-N-(3,4,5-trimethoxyphenyl)-1,3-benzoxazol-2-amine is represented in gold. Yellow dashes in the image represent hydrogen binding interactions.
