# Supplementary material for: Benzyl-Naphthoquinones as Selective Anticancer Agents for Oral Squamous Cell Carcinoma via Apoptosis Induction
Source: Biomedicines. 2026 Mar 26;14(4):757. doi: 10.3390/biomedicines14040757 (PMC13113836; doi:10.3390/biomedicines14040757)

Benzyl-Naphthoquinones as Selective Anticancer Agents for Oral Squamous Cell Carcinoma via Apoptosis Induction

Antonio Mendonça Marconi-Nicolau[1†], Rebeca Gripp de Sá[2†], Caroline Reis Santiago Paschoal[3], Lethícia Andrade de Almeida[2], Gabriel Ouverney[1], Ana Caroline dos Santos Diniz[4], Anamel Blaudt Meira[4], João Pedro da Costa Faria Brunhosa[2], Luiz Carlos da Silva Pinheiro[5], Paula Alvarez Abreu[3], Vinicius Rangel Campos[2*], Bruno K. Robbs[4*]

1 Programa de Pós-Graduação em Ciências Morfológicas, Instituto de Ciências Biomédicas, Universidade Federal do Rio de Janeiro, Fundão, Rio de Janeiro 21941-902, RJ, Brazil. Email: **antoniomarconi@id.uff.br, ouverneygabriel@id.uff.br**

2 Universidade Federal Fluminense, Department of Organic Chemistry, Institute of Chemistry, Campus do Valonguinho, CEP 24020-150, Niterói-RJ, Brazil; Email: **viniciuscampos@id.uff.br, rebecagrippdesa@gmail.com, lethicia.andrade2@gmail.com, joaopedrobrunhosa@id.uff.br, pinheirolcs@gmail.com**

3 Universidade Federal do Rio de Janeiro, Instituto de Biodiversidade e Sustentabilidade NUPEM, CEP 27965-045, Macaé, RJ, Brazil; Email: **abreu_pa@yahoo.com.br**, **carolinesantiago@live.com**

4 Universidade Federal Fluminense, Department of Basic Sciences, Nova Friburgo Health Institute, CEP 28625-650, Nova Friburgo-RJ, Brazil. Email: **brunokr@id.uff.br**, **ana_diniz@id.uff.br, anamelmeira@gmail.com.**

5 Universidade do Estado do Rio de Janeiro - UERJ, Departamento de Ciências, Faculdade de Formação de Professores, São Gonçalo, RJ, Brazil; Email: **pinheirolcs@gmail.com**

[†] These authors have contributed equally to this work.
***** Correspondence: **viniciuscampos@id.uff.br** (V.R.C.); brunokr@id.uff.br (B. K. R.); TEL.: +55 22 98133-3489 (V.R.C..); +55 21 99772-1259 (B. K. R.)

Academic Editor: Firstname Lastname

Received: date
Revised: date
Accepted: date
Published: date

Citation: To be added by editorial staff during production.

Copyright: © 2025 by the authors. Submitted for possible open access publication under the terms and conditions of the Creative Commons Attribution (CC BY) license (https://creativecommons.org/licenses/by/4.0/).

Compounds spectra

Figure S1. IR of 4-(benzylamino)naphthalene-1,2-dione (1)

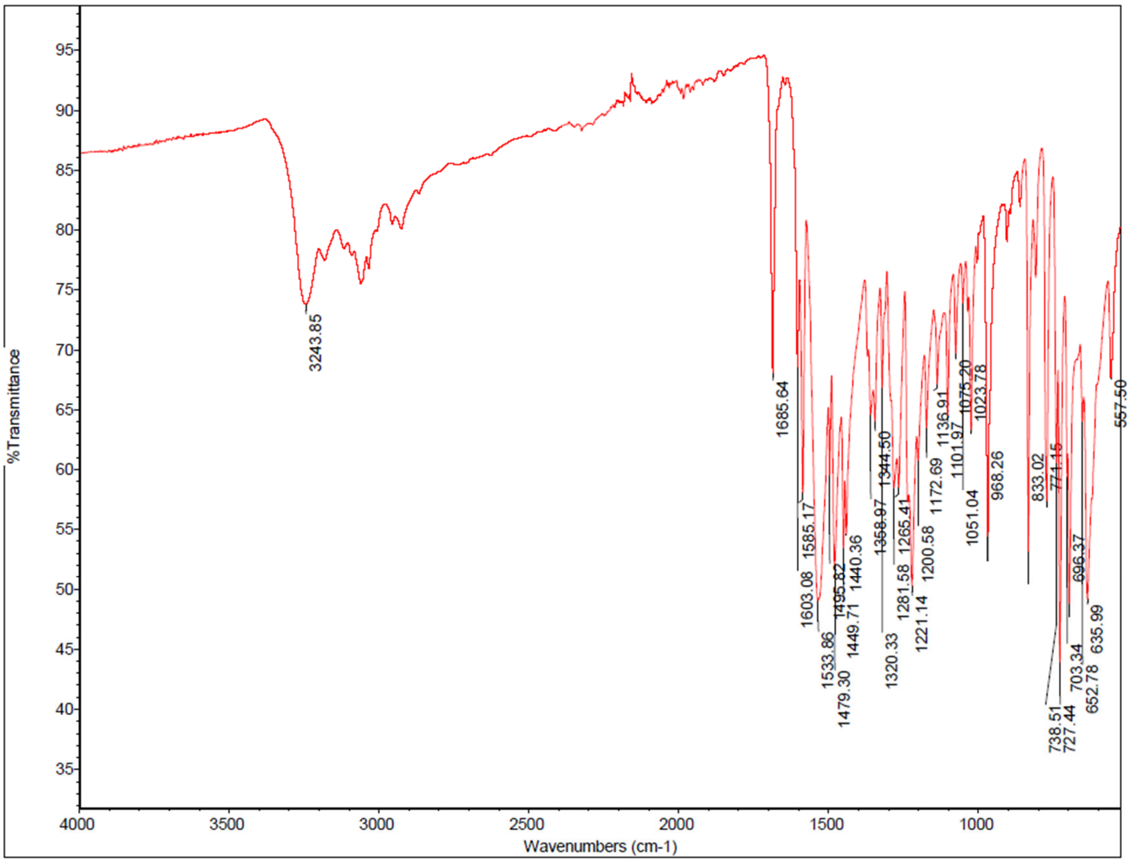

Figure S2. ^{1}H NMR (500 MHz. DMSO-d_6) of 4-(benzylamino)naphthalene-1,2-dione (1)

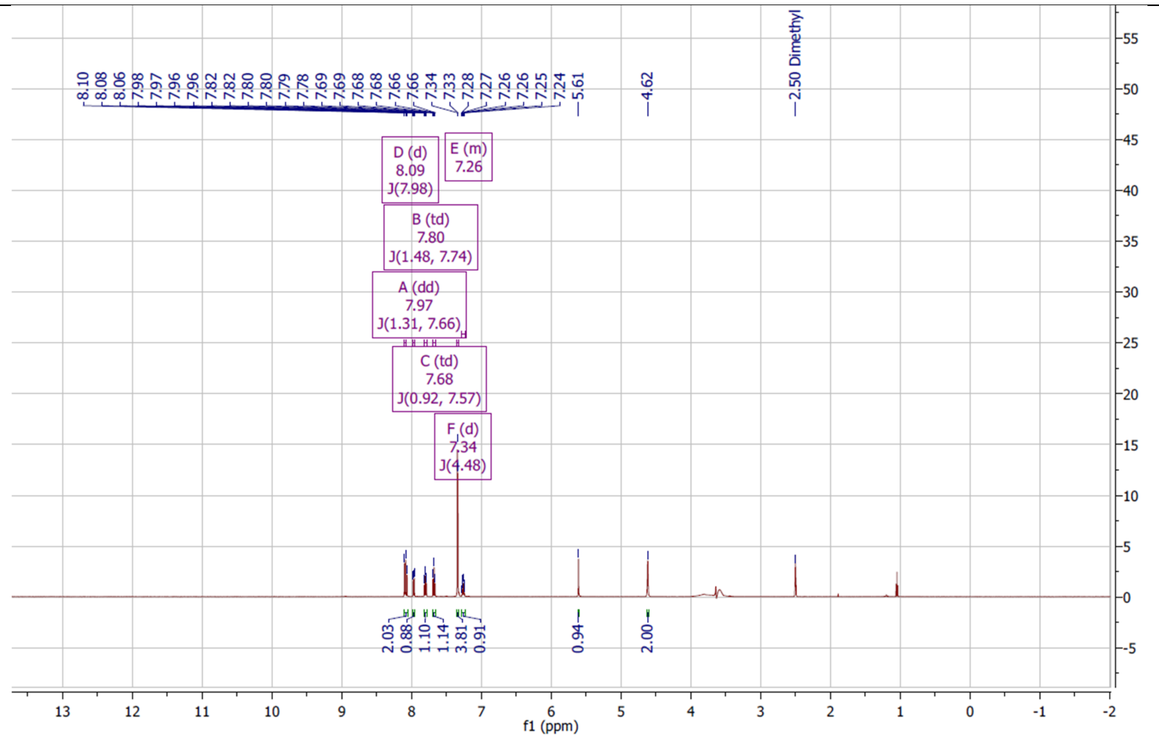

Figure S3. HRMS of 4-(benzylamino)naphthalene-1,2-dione (1)

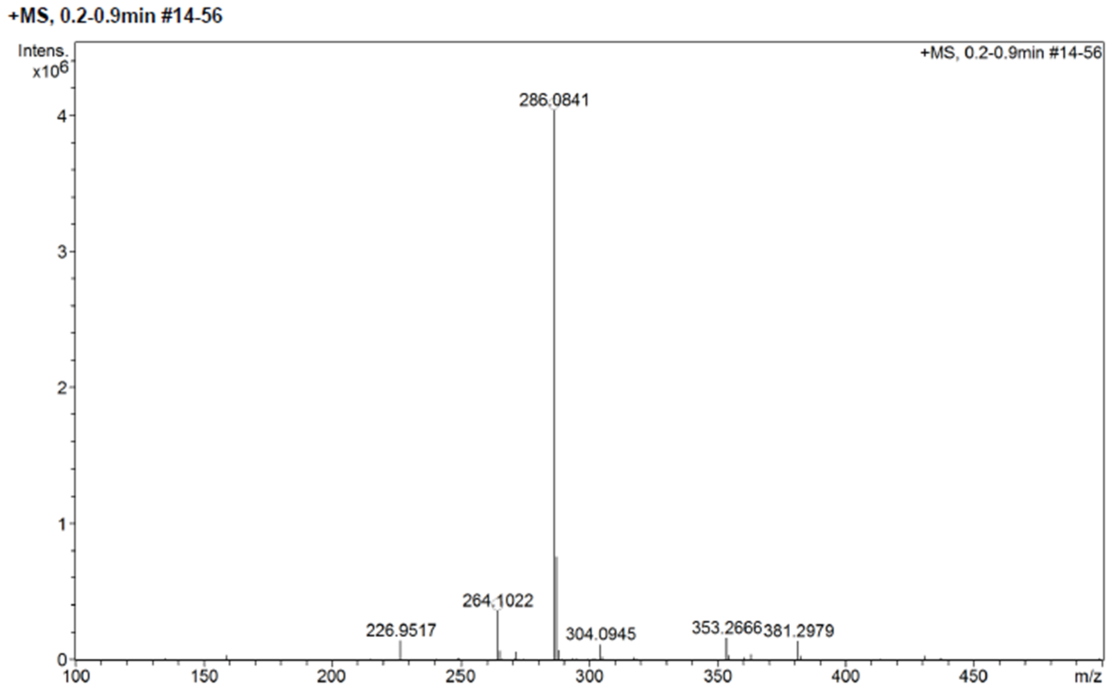

Meas. m/z	#	Ion Formula	m/z	err [ppm]	Mean err [ppm]	rdb	N-Rule	e⁻ Conf	mSigma	Std I	Std Mean m/z	Std I VarNorm	Std m/z Diff	Std Comb Dev
264.102219	1	C17H14NO2	264.101905	-1.2	-0.6	11.5	ok	even	0.4	0.6	n.a.	n.a.	n.a.	n.a.
286.084053	1	C17H13NNaO2	286.083849	-0.7	-0.3	11.5	ok	even	1.5	3.0	n.a.	n.a.	n.a.	n.a.

Figure S4. IR of 4-((4-methylbenzyl)amino)naphthalene-1,2-dione (2)

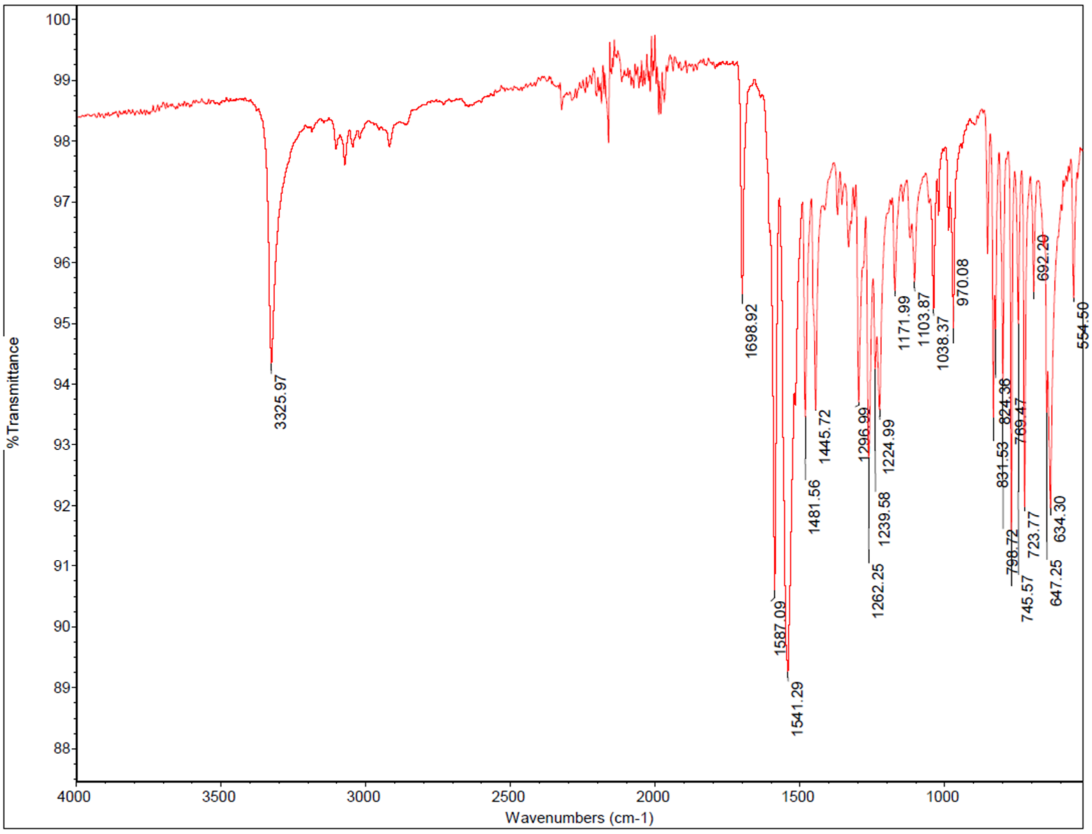

Figure S5. ^{1}H NMR (500 MHz. DMSO-d_6) of 4-((4-methylbenzyl)amino)naphthalene-1,2-dione (2)

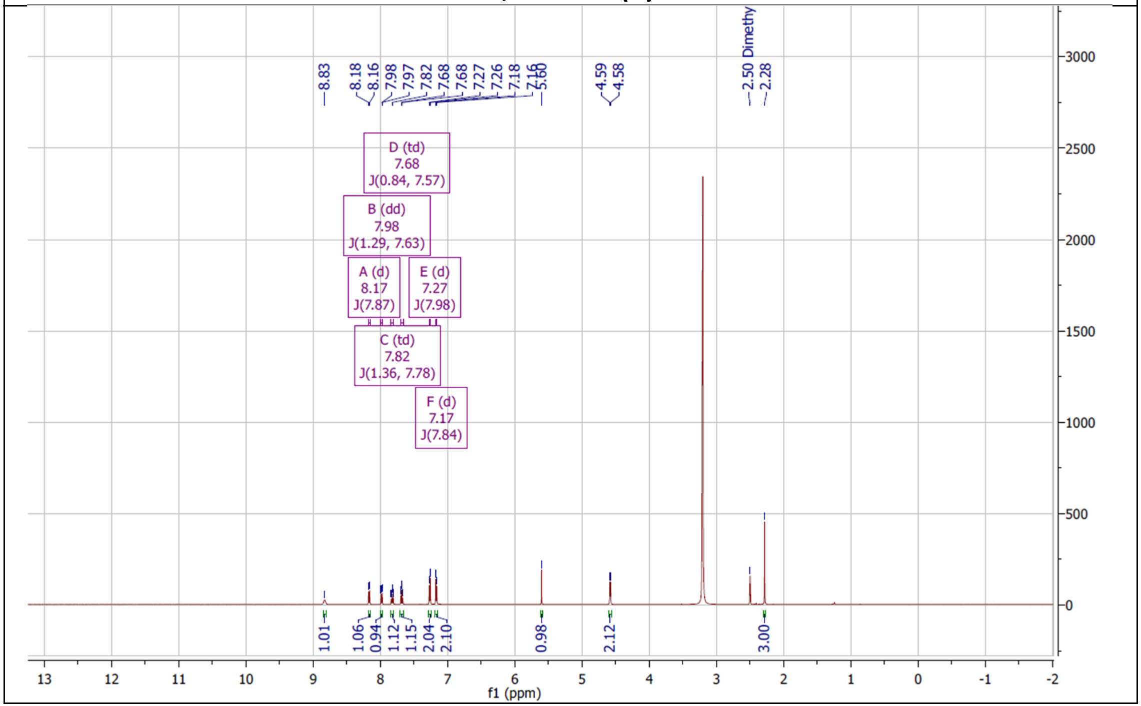

Figure S6. **^{13}C NMR/APT (125 MHz. DMSO-d_6) of 4-((4-methylbenzyl)amino)naphthalene-1,2-dione (2)**

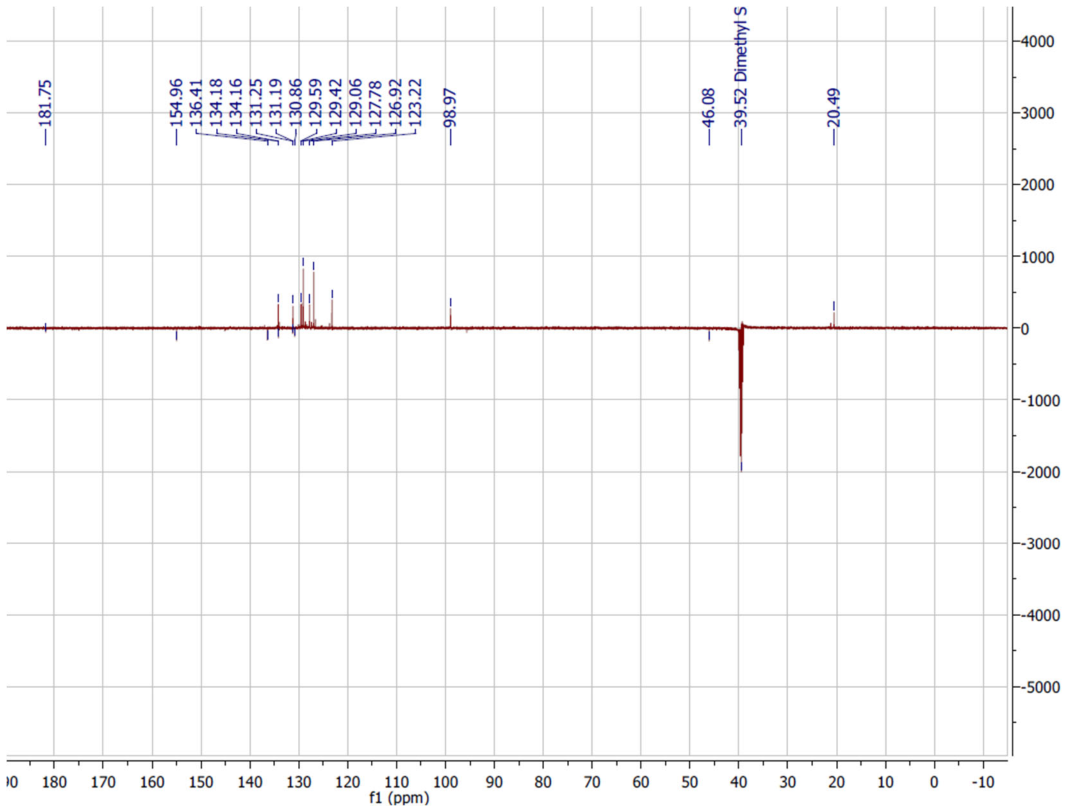

Figure S7. HRMS of 4-((4-methylbenzyl)amino)naphthalene-1,2-dione (2)

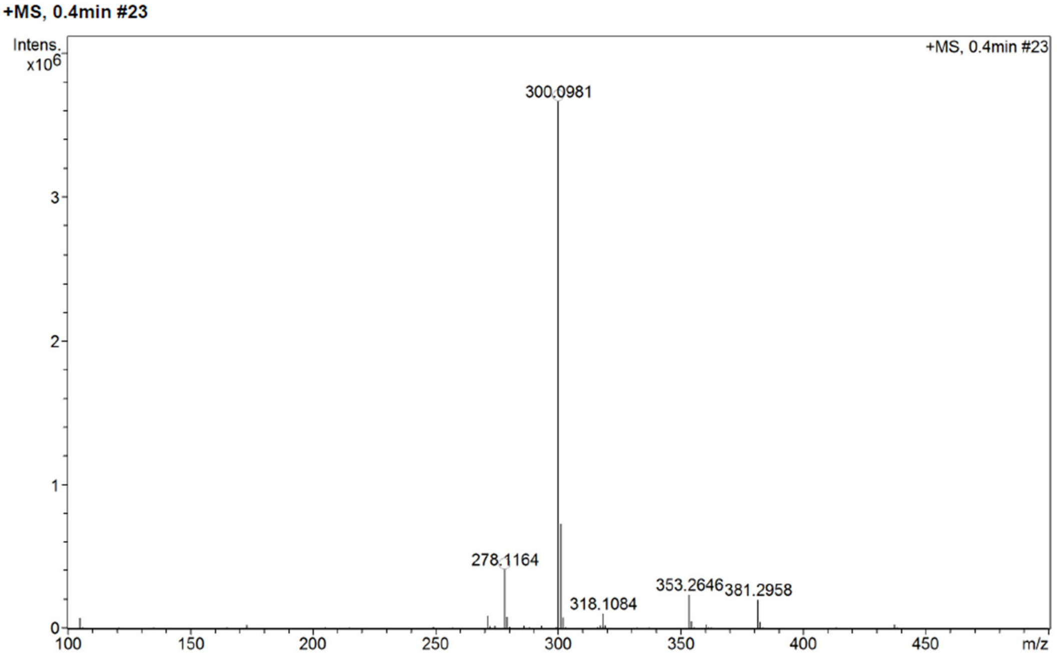

Meas. m/z	#	Ion Formula	m/z	err [ppm]	Mean err [ppm]	rdb	N-Rule	e⁻ Conf	mSigma	Std I	Std Mean m/z	Std I VarNorm	Std m/z Diff	Std Comb Dev
278.116382	1	$C_{18}H_{16}NO_2$	278.117555	4.2	3.6	11.5	ok	even	0.7	1.0	n.a.	n.a.	n.a.	n.a.
300.098076	1	$C_{18}H_{15}NNaO_2$	300.099499	4.7	5.1	11.5	ok	even	1.2	2.5	n.a.	n.a.	n.a.	n.a.
	2	$C_{14}H_{11}N_7Na$	300.096814	-4.2	-5.4	12.5	ok	even	13.0	24.3	n.a.	n.a.	n.a.	n.a.

Figure S8. IR of 4-((4-methoxybenzyl)amino)naphthalene-1,2-dione (3)

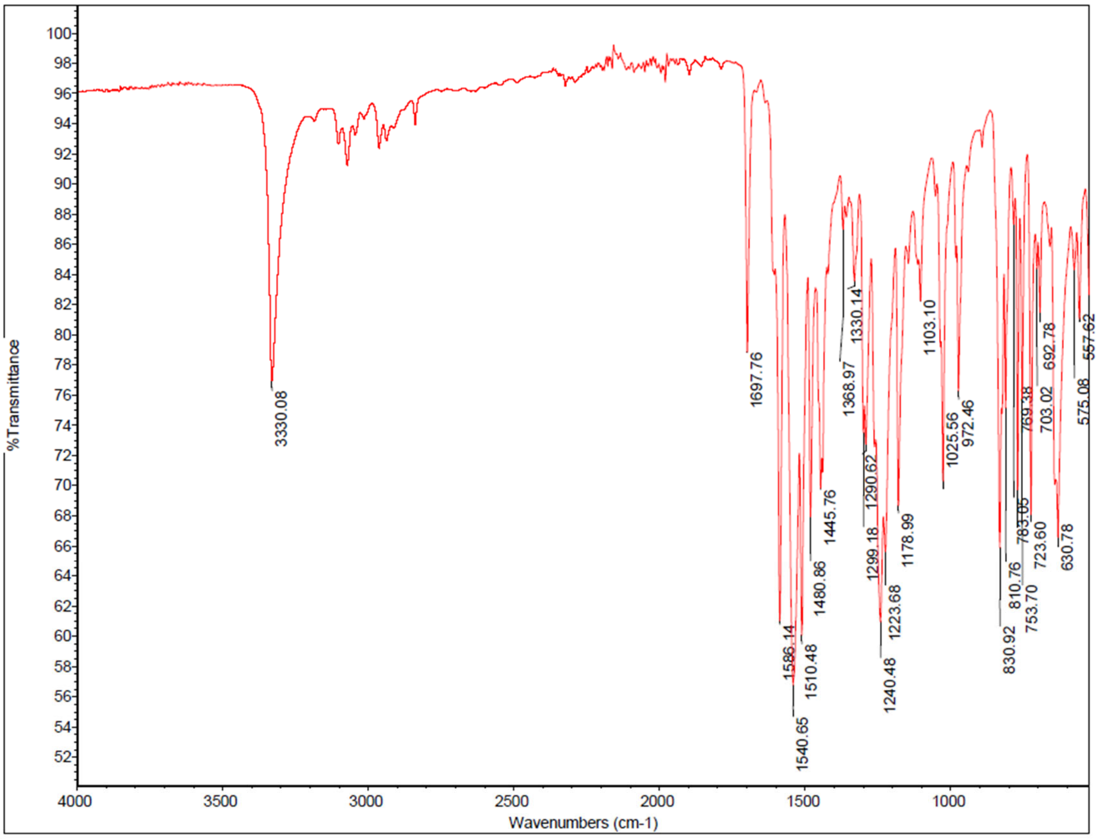

Figure S9. ^{1}H NMR (500 MHz. MeOD) of 4-((4-methoxybenzyl)amino)naphthalene-1,2-dione (3)

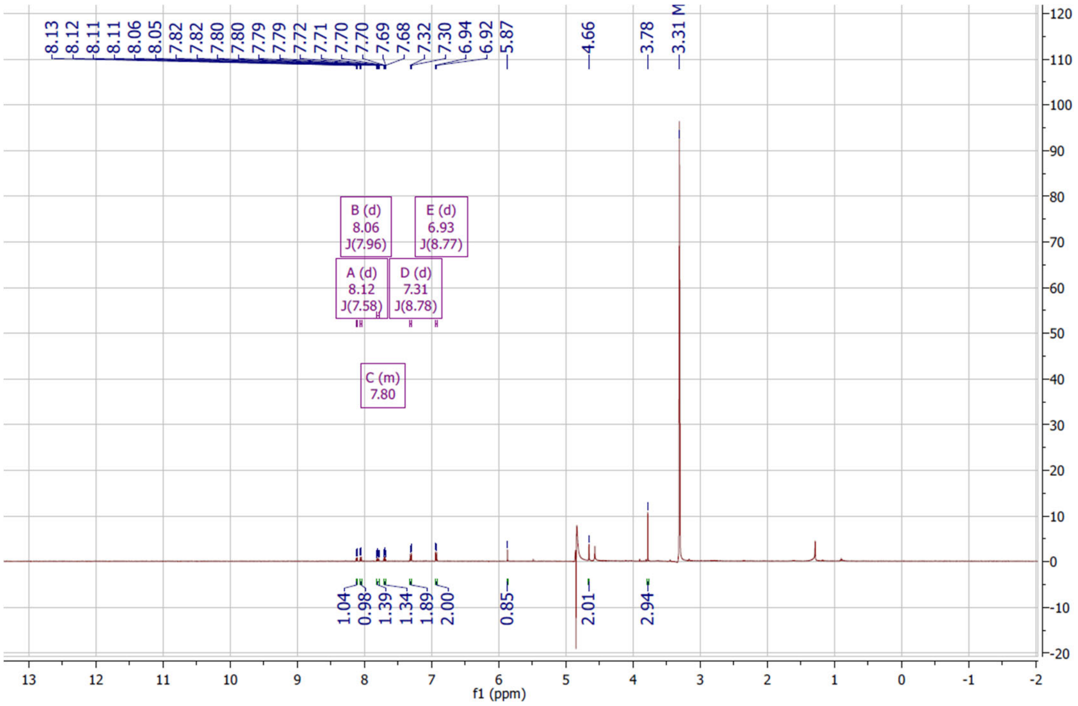

Figure S10. HRMS of 4-((4-methoxybenzyl)amino)naphthalene-1,2-dione (3)

+MS, 0.0-0.3min #2-19

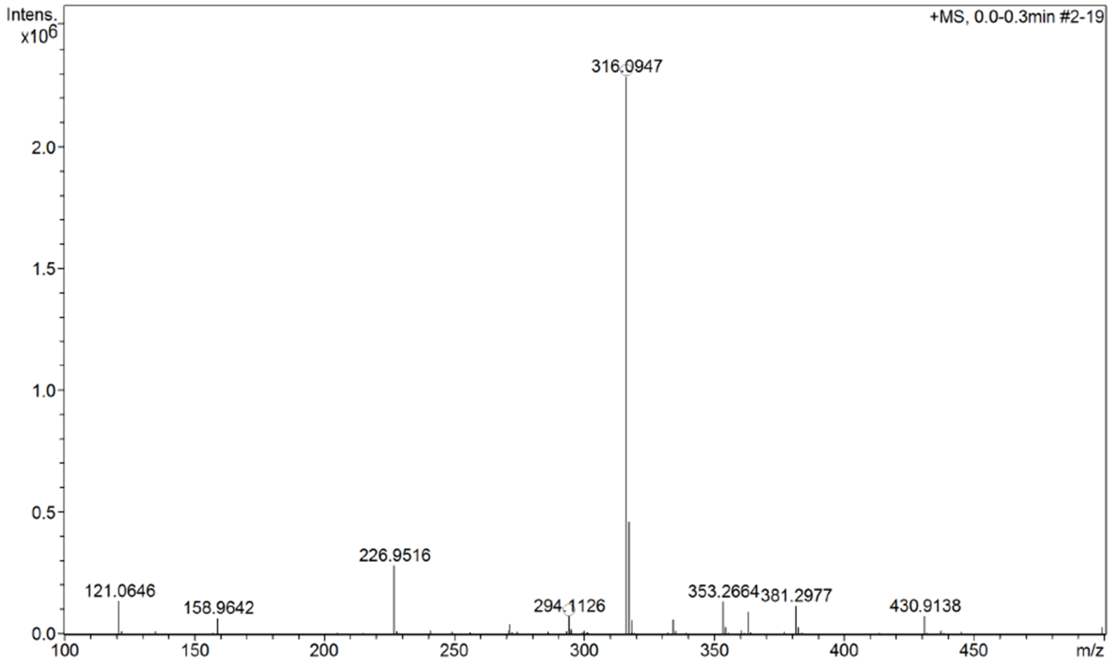

Meas. m/z	#	Ion Formula	m/z	err [ppm]	Mean err [ppm]	rdb	N-Rule	e⁻ Conf	mSigma	Std I	Std Mean m/z	Std I VarNorm	Std m/z Diff	Std Comb Dev
294.112584	1	C18H16NO3	294.112470	-0.4	-0.3	11.5	ok	even	3.5	6.4	n.a.	n.a.	n.a.	n.a.
316.094709	1	C18H15NNaO3	316.094414	-0.9	-0.6	11.5	ok	even	0.6	1.0	n.a.	n.a.	n.a.	n.a.

Figure S11. IR of 4-((4-chlorobenzyl)amino)naphthalene-1,2-dione (4)

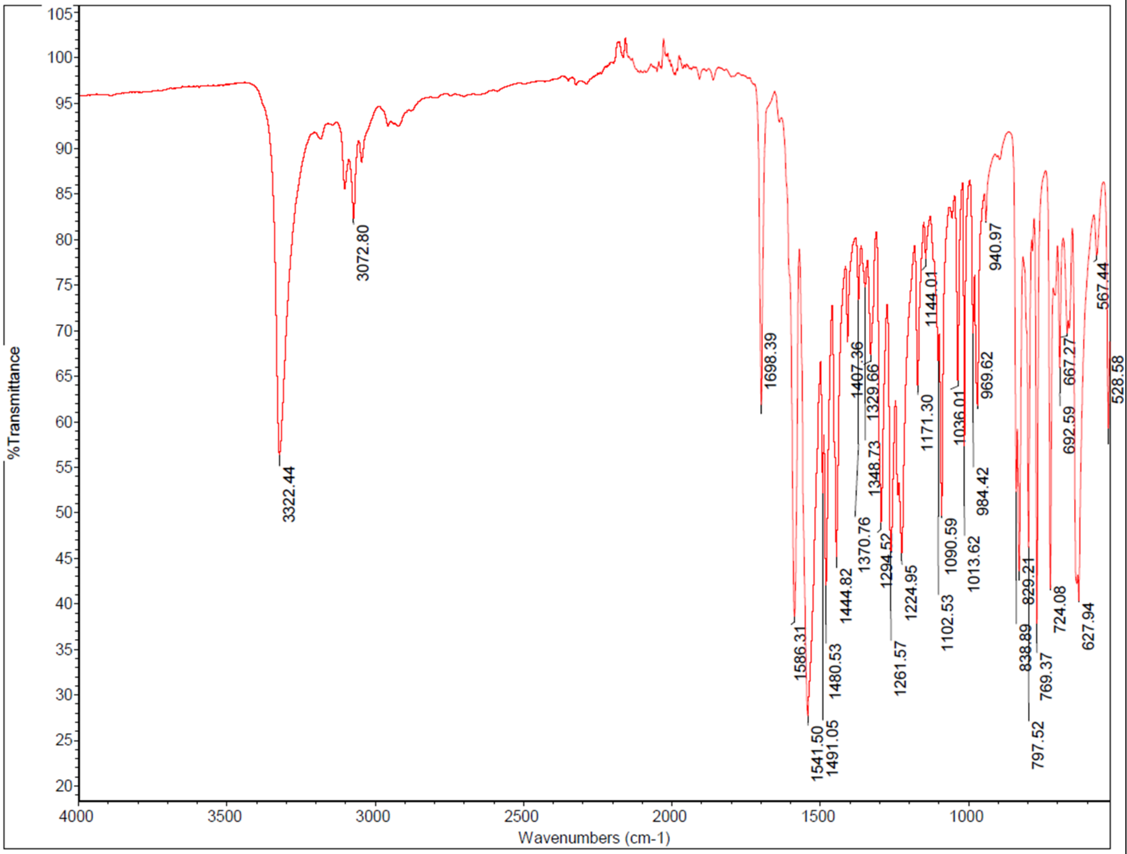

Figure S12. ^{1}**H NMR (500 MHz. DMSO-*d*$_6$) of 4-((4-chlorobenzyl)amino)naphthalene-1,2-dione (4)**

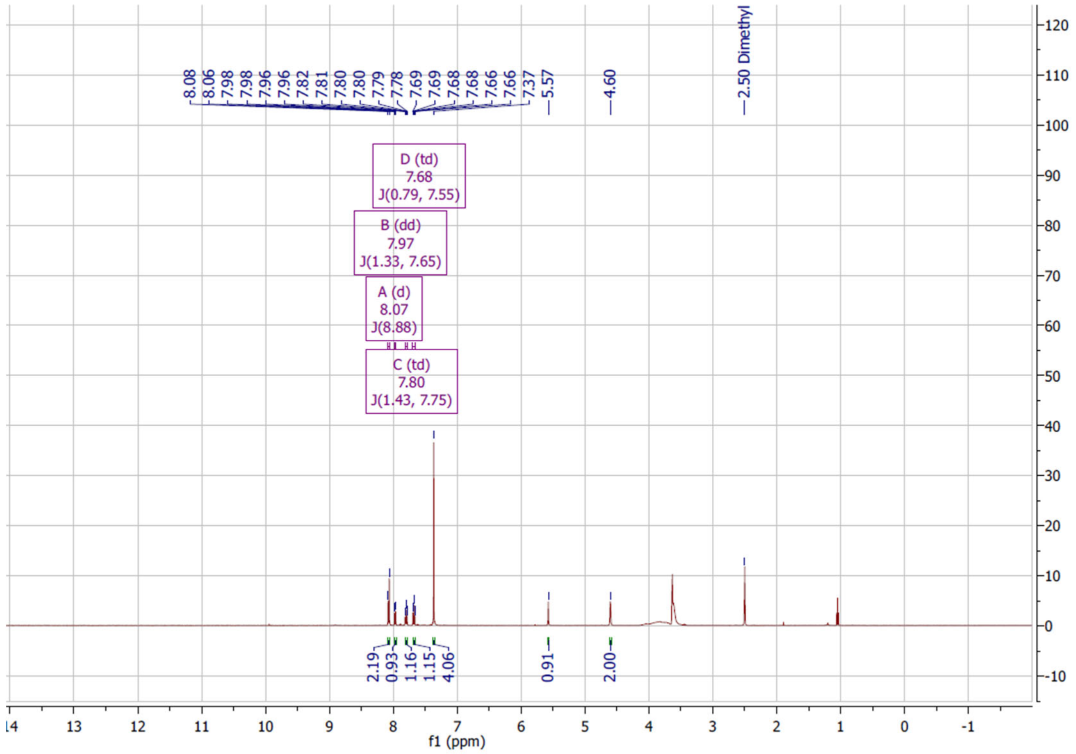

Figure S13. HRMS of 4-((4-chlorobenzyl)amino)naphthalene-1,2-dione (4)

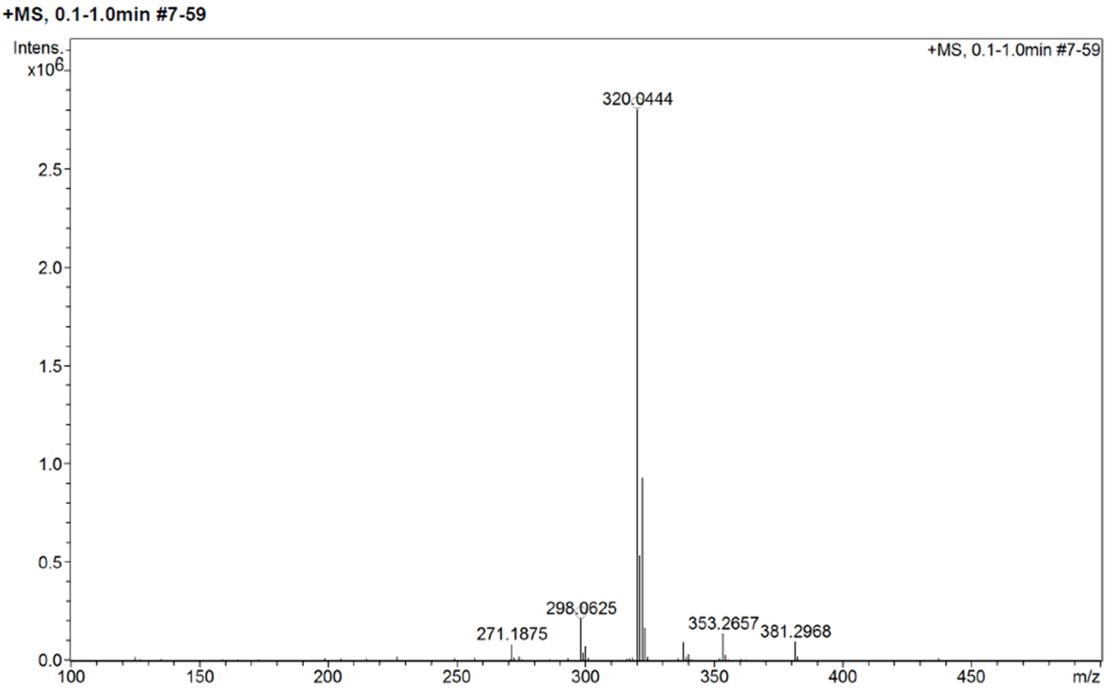

Meas. m/z	#	Ion Formula	m/z	err [ppm]	Mean err [ppm]	rdb	N-Rule	e⁻ Conf	mSigma	Std I	Std Mean m/z	Std I VarNorm	Std m/z Diff	Std Comb Dev
298.062524	1	C17H13ClNO2	298.062933	1.4	1.6	11.5	ok	even	2.0	3.0	n.a.	n.a.	n.a.	n.a.
320.044362	1	C17H12ClNNaO2	320.044877	1.6	1.8	11.5	ok	even	4.9	6.5	n.a.	n.a.	n.a.	n.a.

Figure S14. IR of 2-(benzylamino)naphthalene-1,4-dione (5)

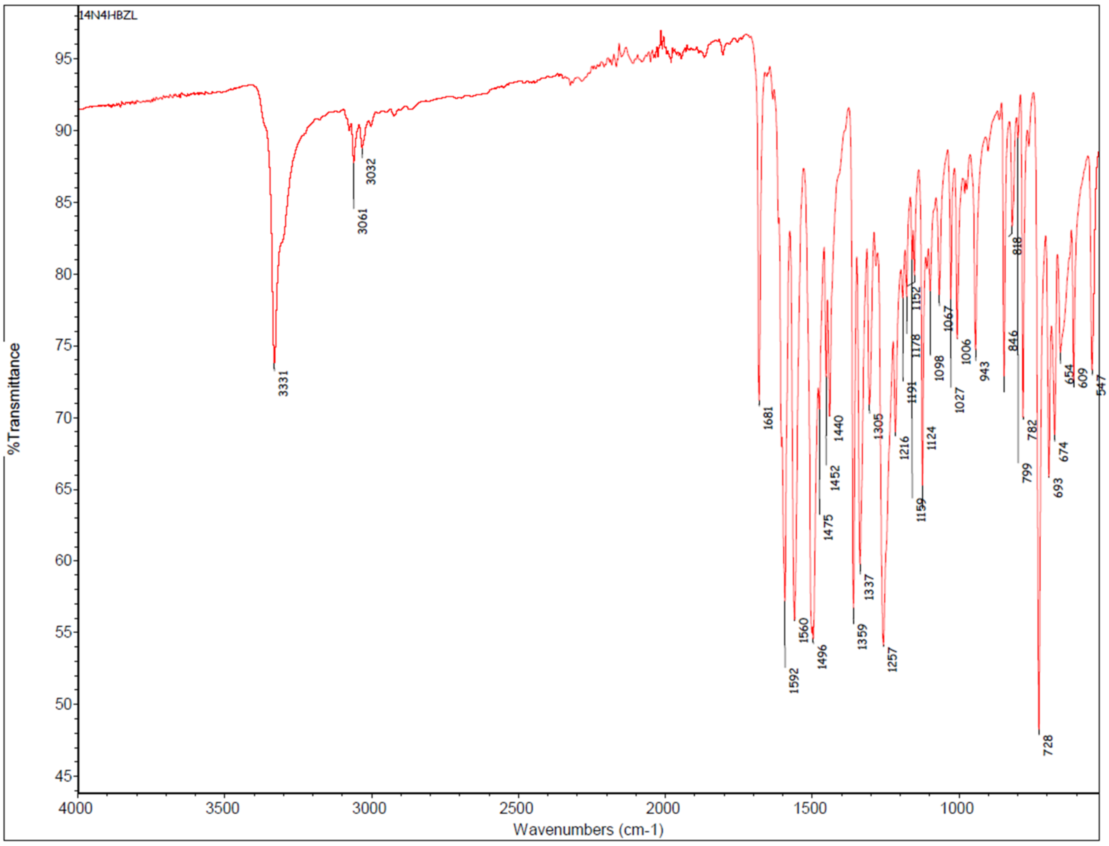

Figure S15. ^{1}H NMR (500 MHz. $CDCl_3$) of 2-(benzylamino)naphthalene-1,4-dione (5)

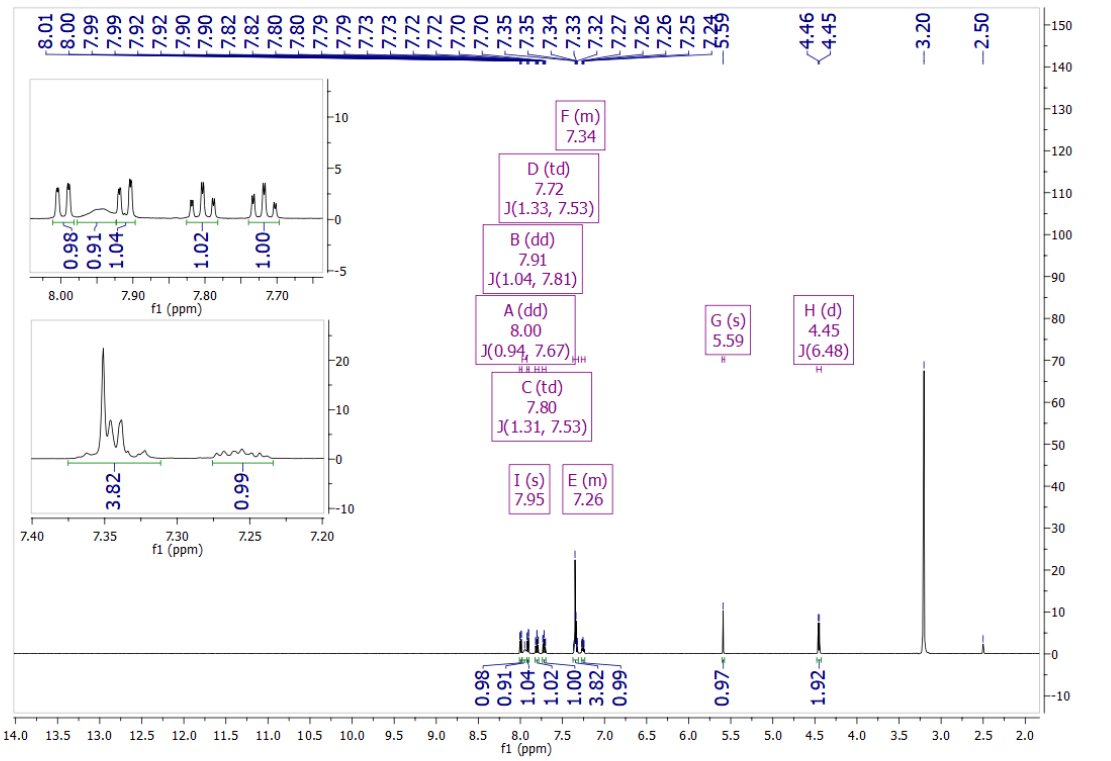

Figure S16. HRMS of 2-(benzylamino)naphthalene-1,4-dione (5)

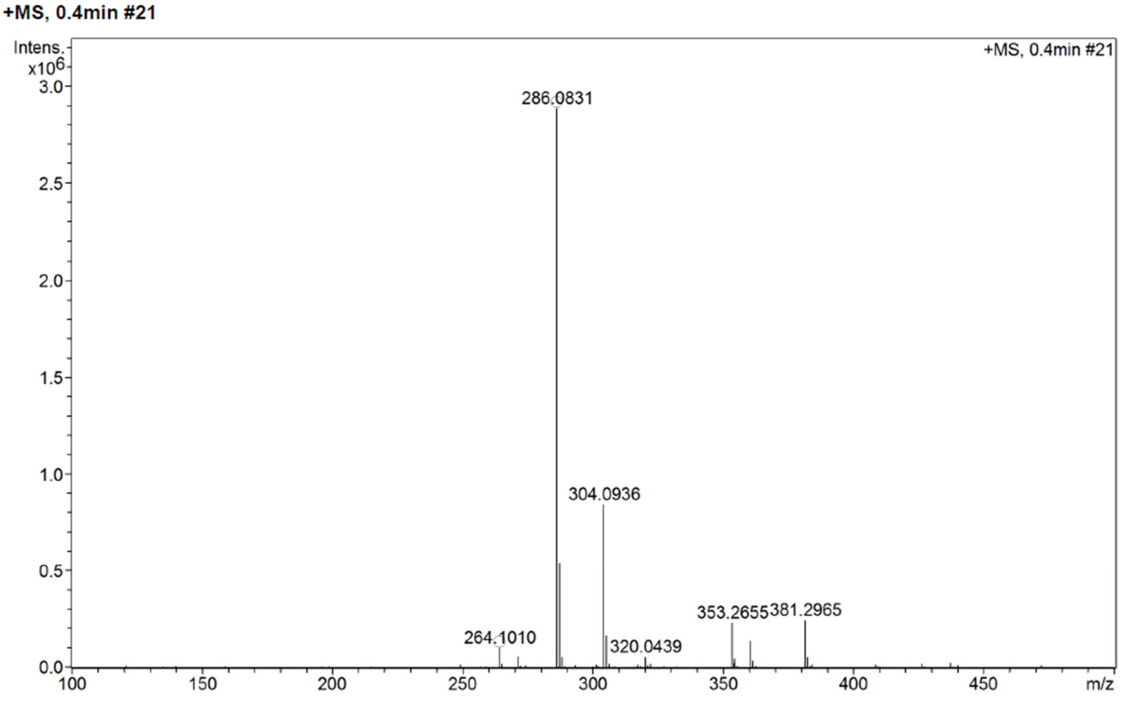

Meas. m/z	#	Ion Formula	m/z	err [ppm]	Mean err [ppm]	rdb	N-Rule	e⁻ Conf	mSigma	Std I	Std Mean m/z	Std I VarNorm	Std m/z Diff	Std Comb Dev
264.100991	1	C17H14NO2	264.101905	3.5	-0.6	11.5	ok	even	3.0	5.3	n.a.	n.a.	n.a.	n.a.
286.083095	1	C17H13NNaO2	286.083849	2.6	2.7	11.5	ok	even	1.4	2.9	n.a.	n.a.	n.a.	n.a.

Figure S17. IR of 2-((4-methylbenzyl)amino)naphthalene-1,4-dione (6)

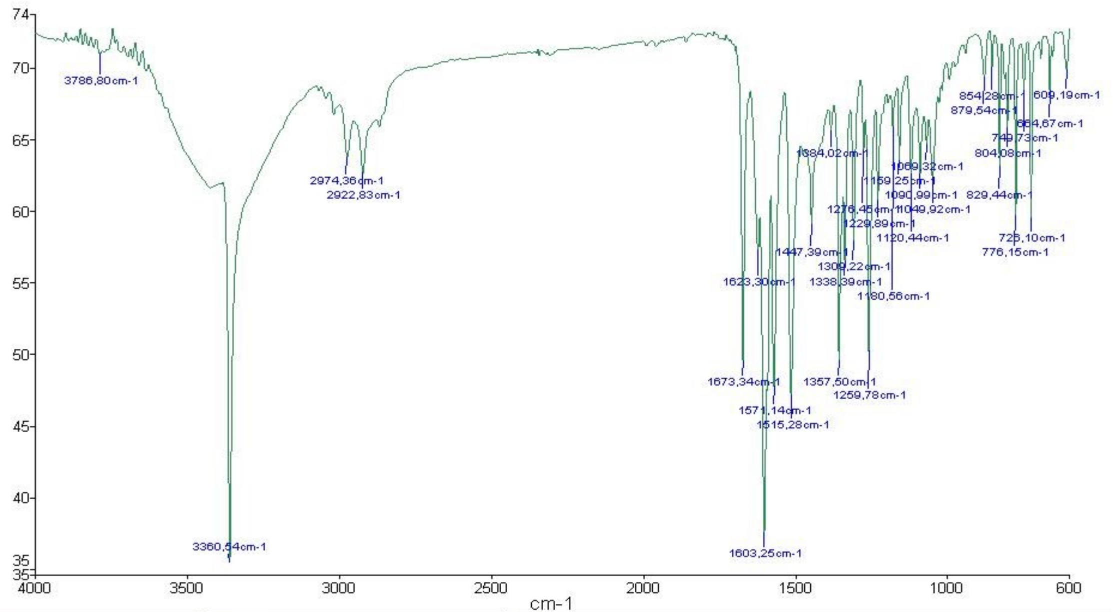

Figure S18. ^{1}H NMR (500 MHz. $CDCl_3$) of 2-((4-methylbenzyl)amino)naphthalene-1,4-dione (6)

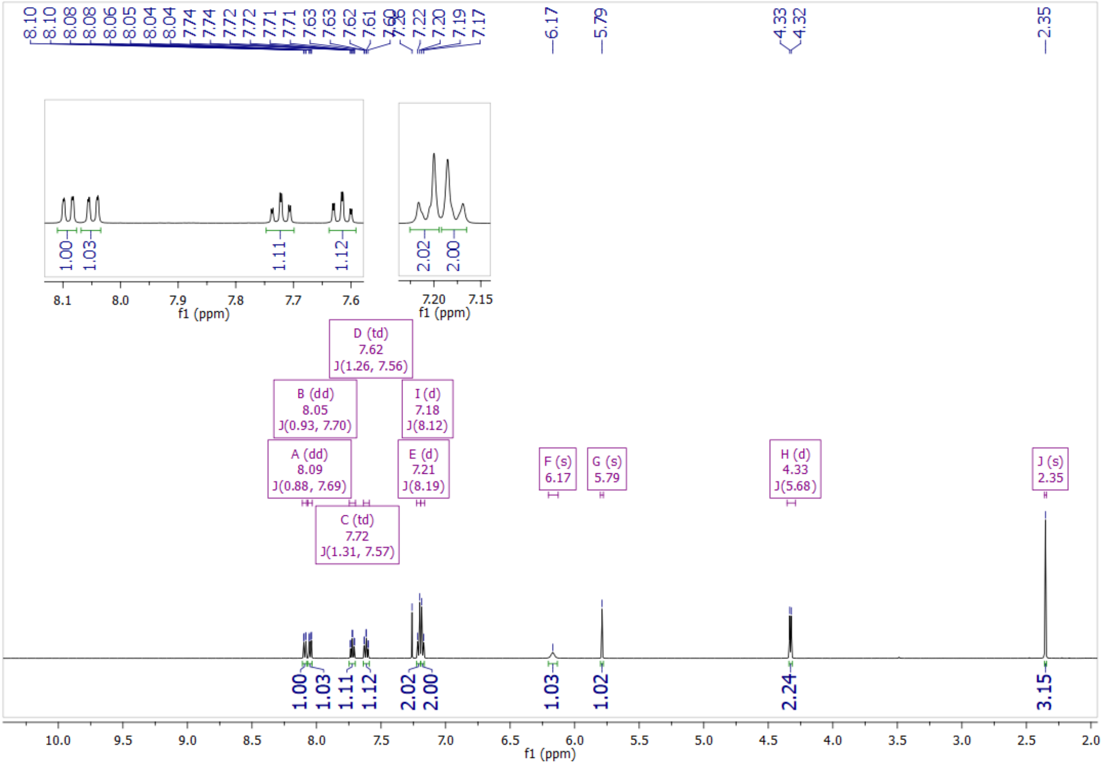

Figure S19. HRMS of 2-((4-methylbenzyl)amino)naphthalene-1,4-dione (6)

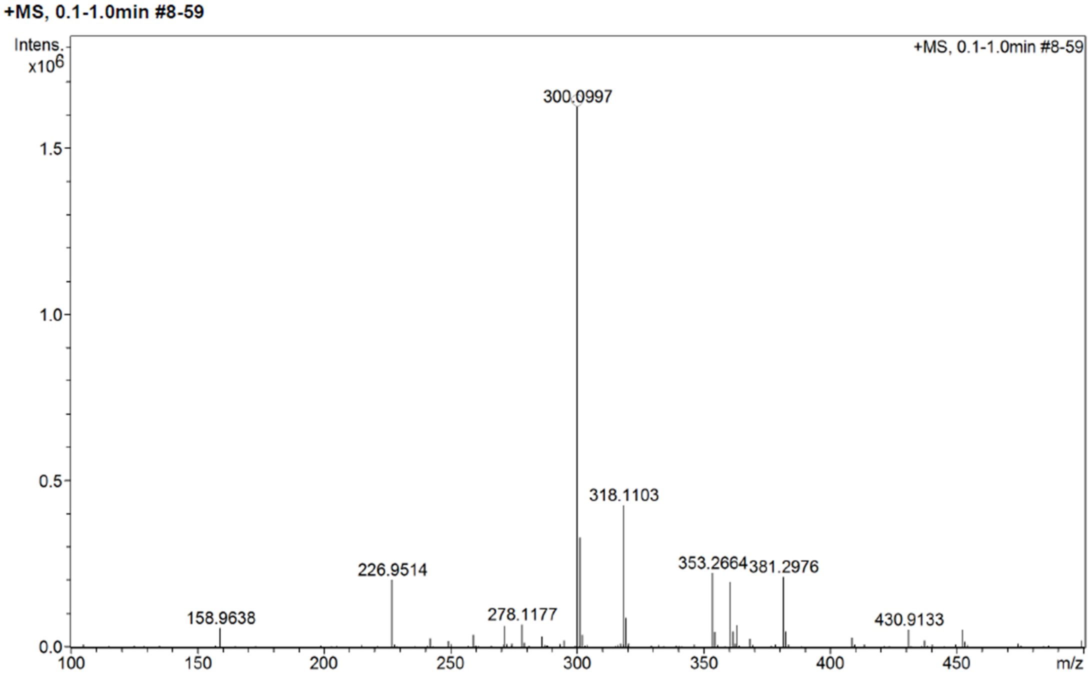

Meas. m/z	#	Ion Formula	m/z	err [ppm]	Mean err [ppm]	rdb	N-Rule	e⁻ Conf	mSigma	Std I	Std Mean m/z	Std I VarNorm	Std m/z Diff	Std Comb Dev
300.099716	1	C18H15NNaO2	300.099499	-0.7	-1.1	11.5	ok	even	2.2	3.5	n.a.	n.a.	n.a.	n.a.

Figure S20. IR of 2-((4-methoxybenzyl)amino)naphthalene-1,4-dione (7)

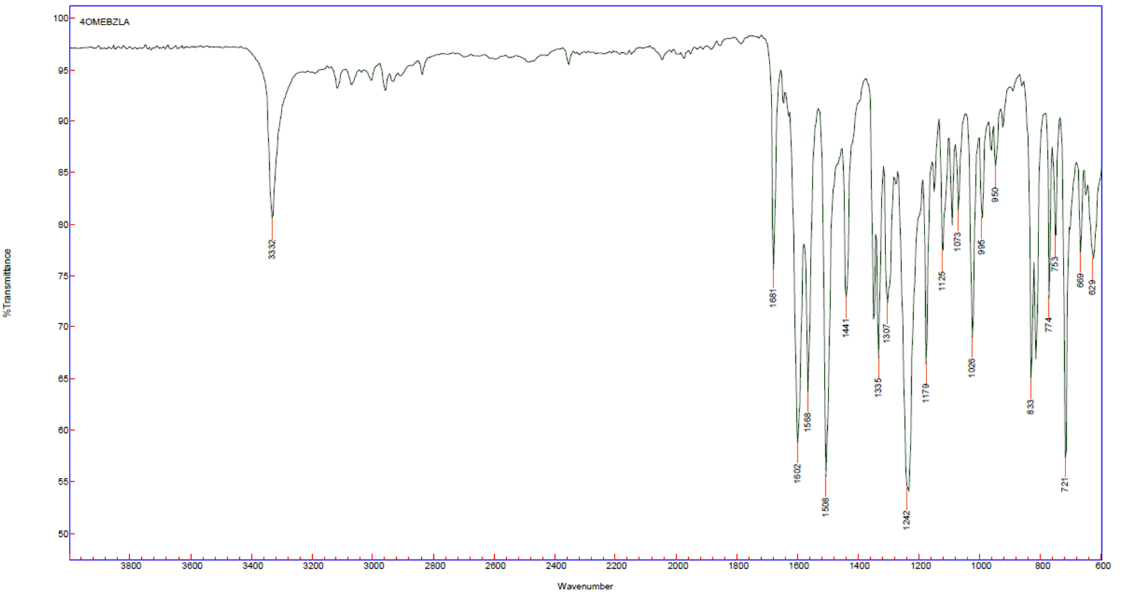

Figure S21. ^{1}H NMR (500 MHz. $CDCl_3$) of 2-((4-methoxybenzyl)amino)naphthalene-1,4-dione (7)

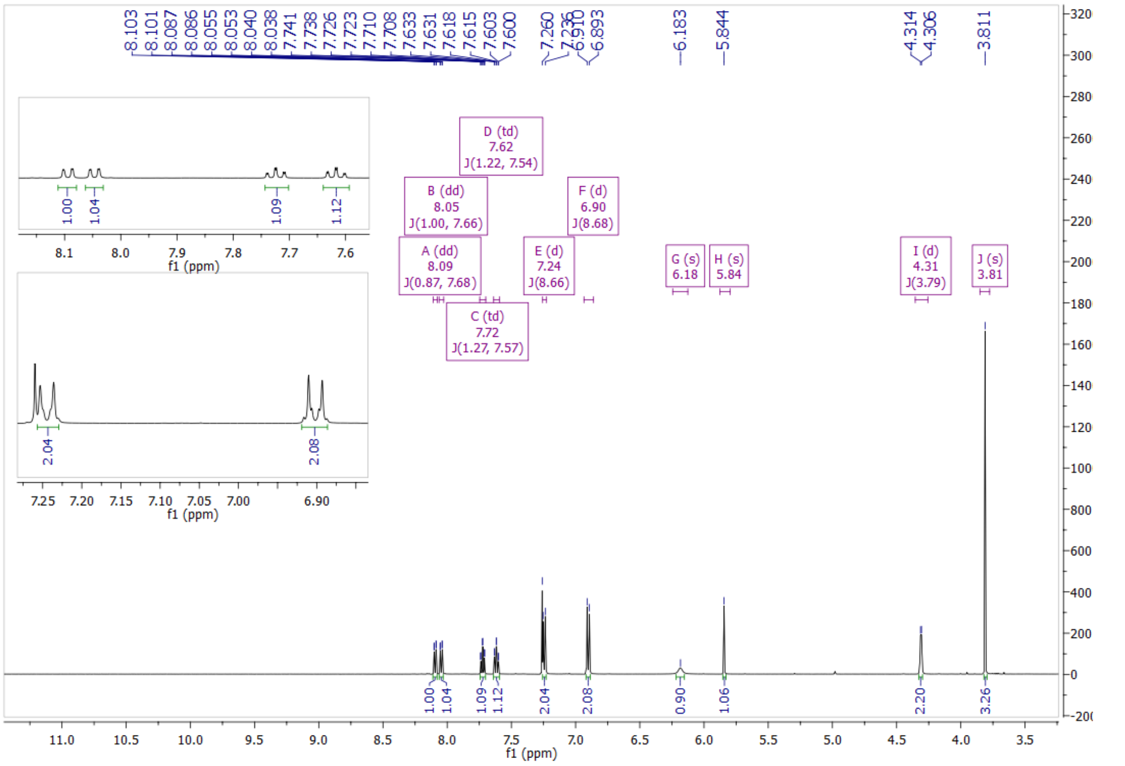

Figure S22. HRMS of 2-((4-methoxybenzyl)amino)naphthalene-1,4-dione (7)

+MS, 0.2-1.0min #11-59

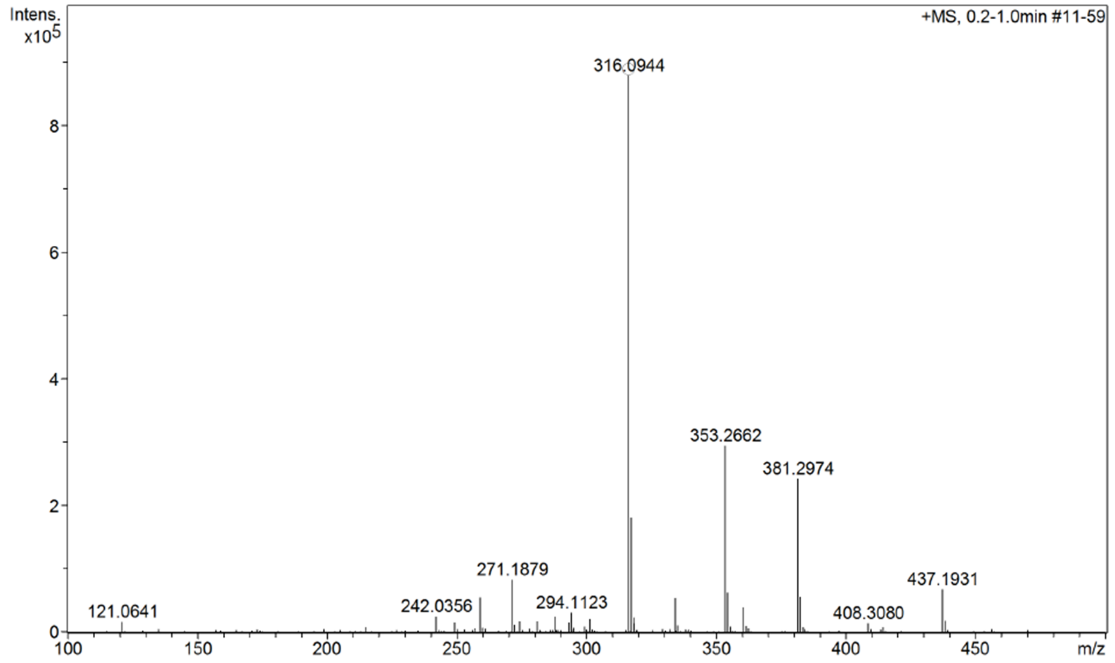

Meas. m/z	#	Ion Formula	m/z	err [ppm]	Mean err [ppm]	rdb	N-Rule	e⁻ Conf	mSigma	Std I	Std Mean m/z	Std I VarNorm	Std m/z Diff	Std Comb Dev
316.094441	1	C18H15NNaO3	316.094414	-0.1	-0.3	11.5	ok	even	3.3	5.8	n.a.	n.a.	n.a.	n.a.

Figure S23. IR of 2-((4-chlorobenzyl)amino)naphthalene-1,4-dione (8)

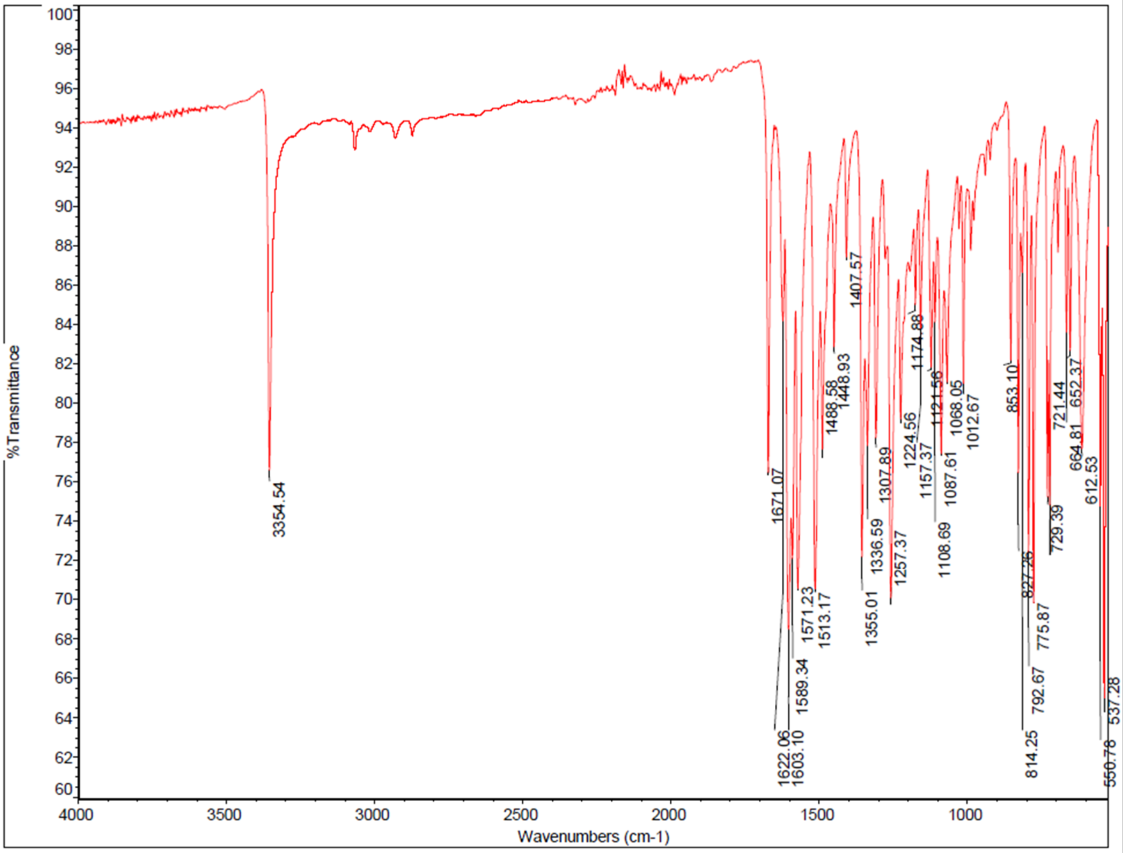

Figure S24. ^{1}H NMR (500 MHz. $CDCl_3$) of 2-((4-chlorobenzyl)amino)naphthalene-1,4-dione (8)

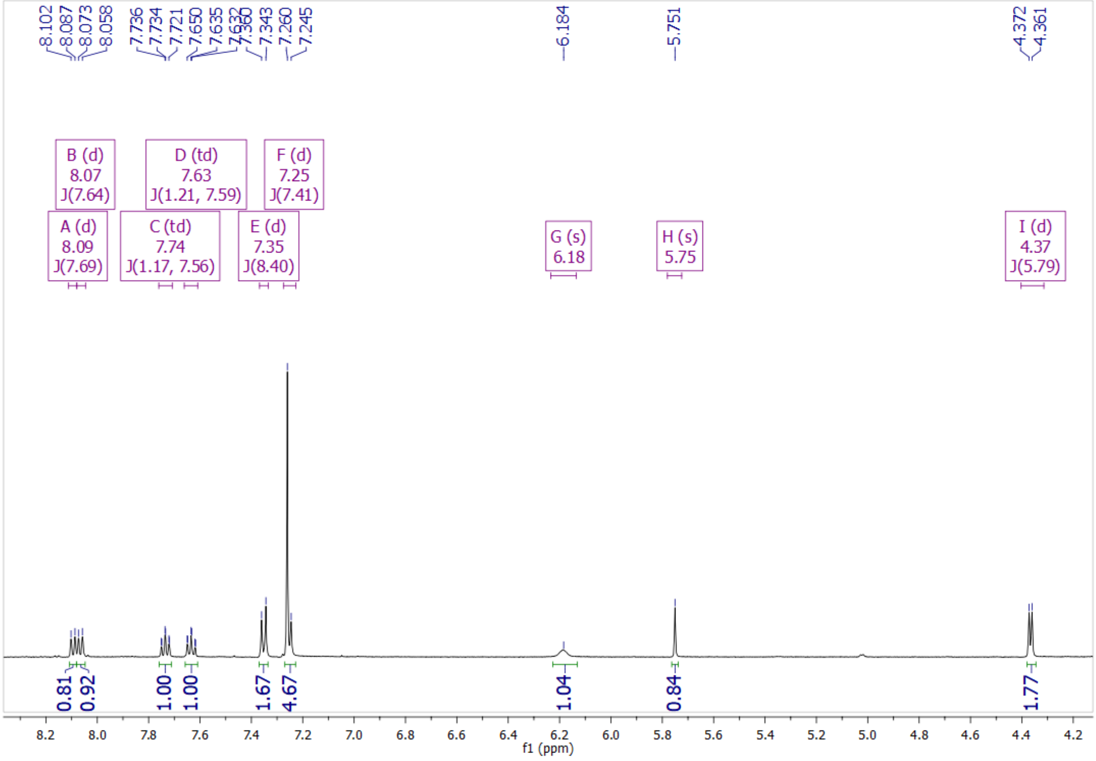

Figure S25. HRMS of 2-((4-chlorobenzyl)amino)naphthalene-1,4-dione (8)

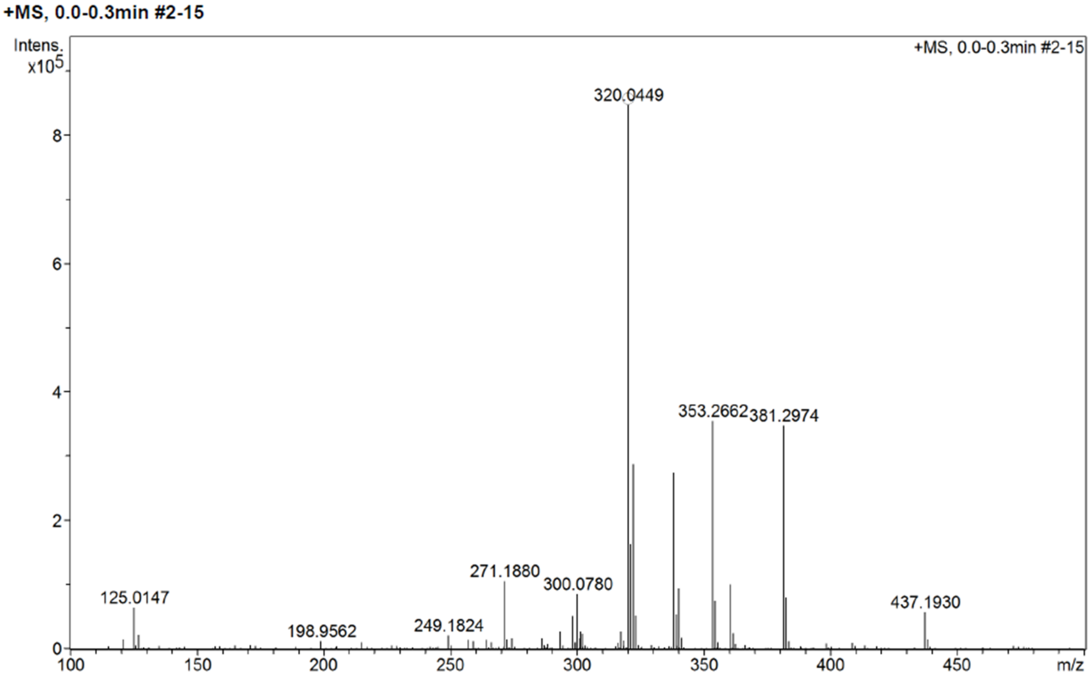

Meas. m/z	#	Ion Formula	m/z	err [ppm]	Mean err [ppm]	rdb	N-Rule	e⁻ Conf	mSigma	Std I	Std Mean m/z	Std I VarNorm	Std m/z Diff	Std Comb Dev
320.044904	1	C17H12ClNNaO2	320.044877	-0.1	-0.0	11.5	ok	even	2.1	2.8	n.a.	n.a.	n.a.	n.a.

Figure S26. Chemical structure of Couma.6e, a previously described naphthoquinone–triazole–coumarin hybrid used in this study as a positive control for autophagy induction. Chemical name: 2-methyl-3-((4-(((2-oxo-2H-chromen-4-yl)oxy)methyl)-1H-1,2,3-triazol-1-yl)(phenyl)methyl)-naphthalene-1,4-dione (Couma. 6e).

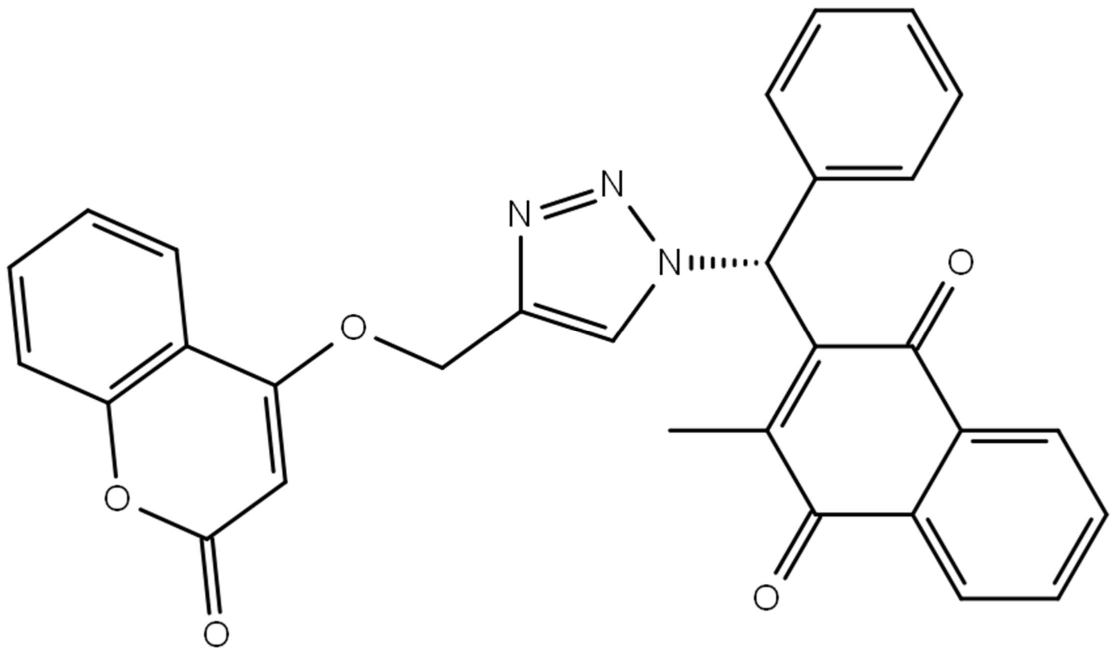

Figure S27. Molecular docking of the compound 1, the drug doxorubicin and the co-crystallized ligand etoposide in the active site of topoisomerase IIα-DNA. Compound 1 is represented in cyan, drug doxorubicin is represented in yellow, the co-crystallized ligand etoposide is represented in orange. Yellow dashes in the image represent hydrogen binding interactions and pink triangles represent ππ-stacking interactions.

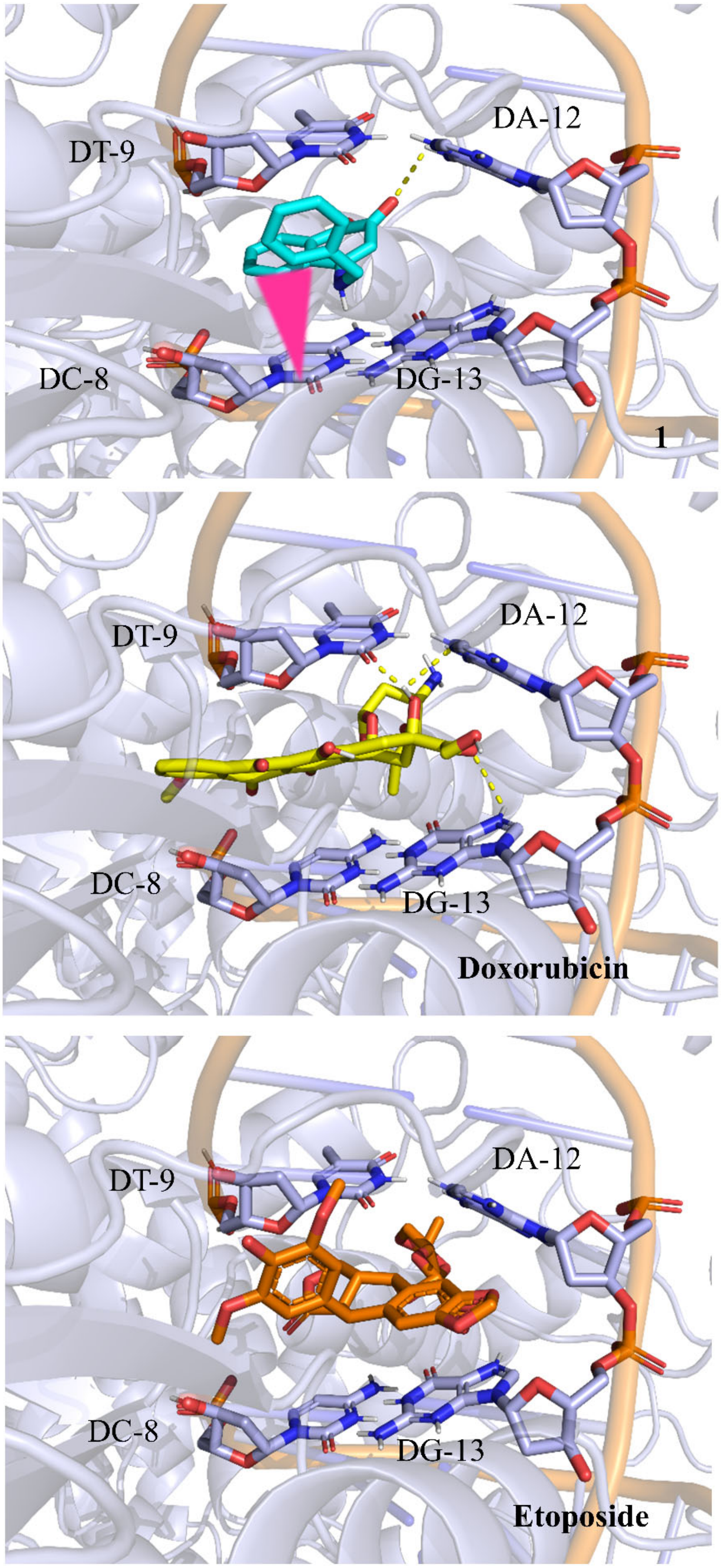

Figure S28. Molecular docking of the compound 1, the drug doxorubicin and the co-crystallized ligand etoposide in the active site of topoisomerase IIβ-DNA. Compound 1 is represented in cyan, drug doxorubicin is represented in yellow, the co-crystallized ligand etoposide is represented in green. Yellow dashes in the image represent hydrogen binding interactions and pink triangles represent ππ-stacking interactions.

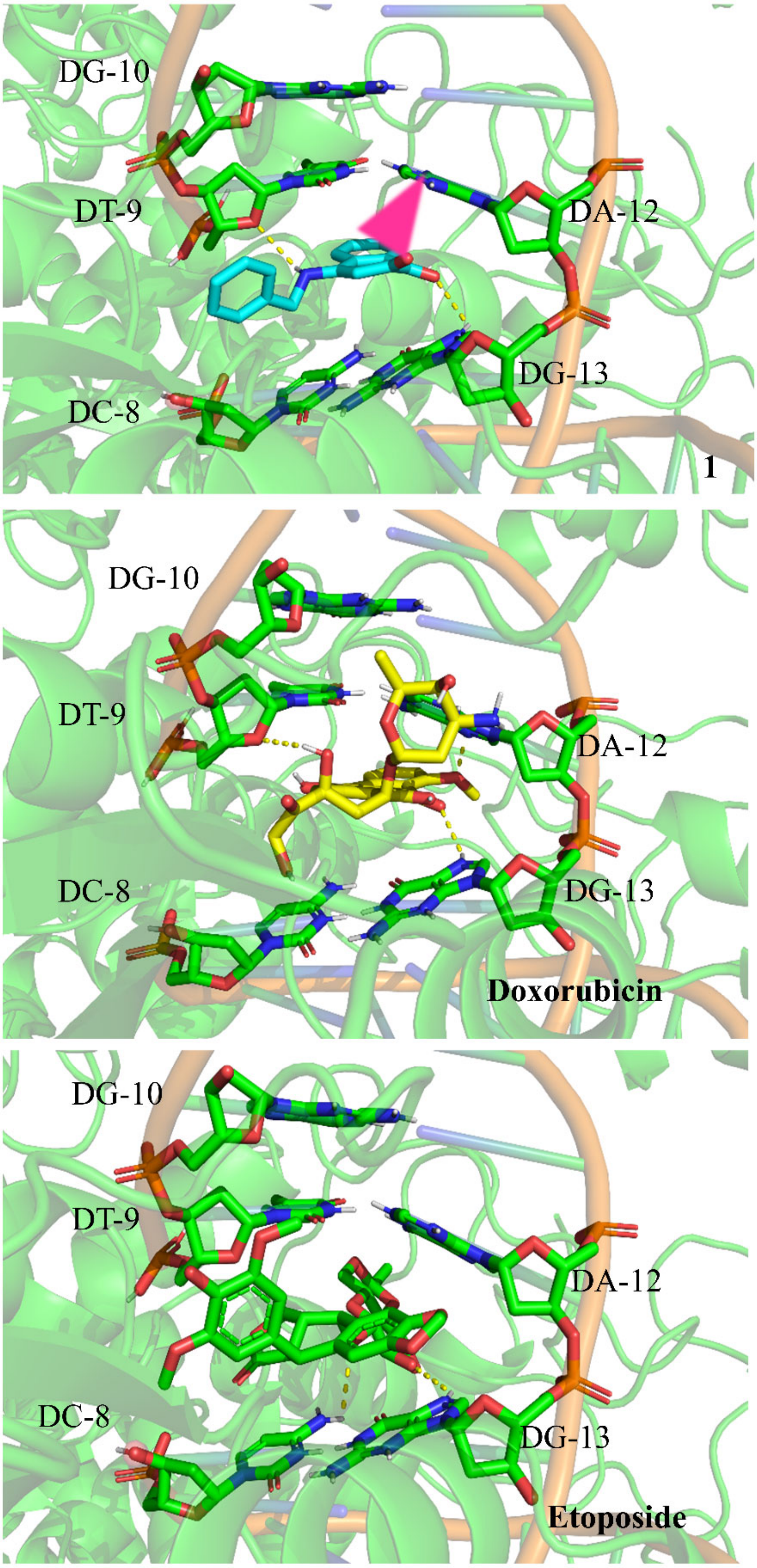

Figure S29. Molecular docking of the compound 1, the inhibitor lapachol and ATP in the active site of PKM2. Compound 1 is represented in cyan, lapachol is represented in pink, and ATP is represented in green. Yellow dashes in the image represent hydrogen binding interactions and pink triangles represent π-π-stacking interactions.

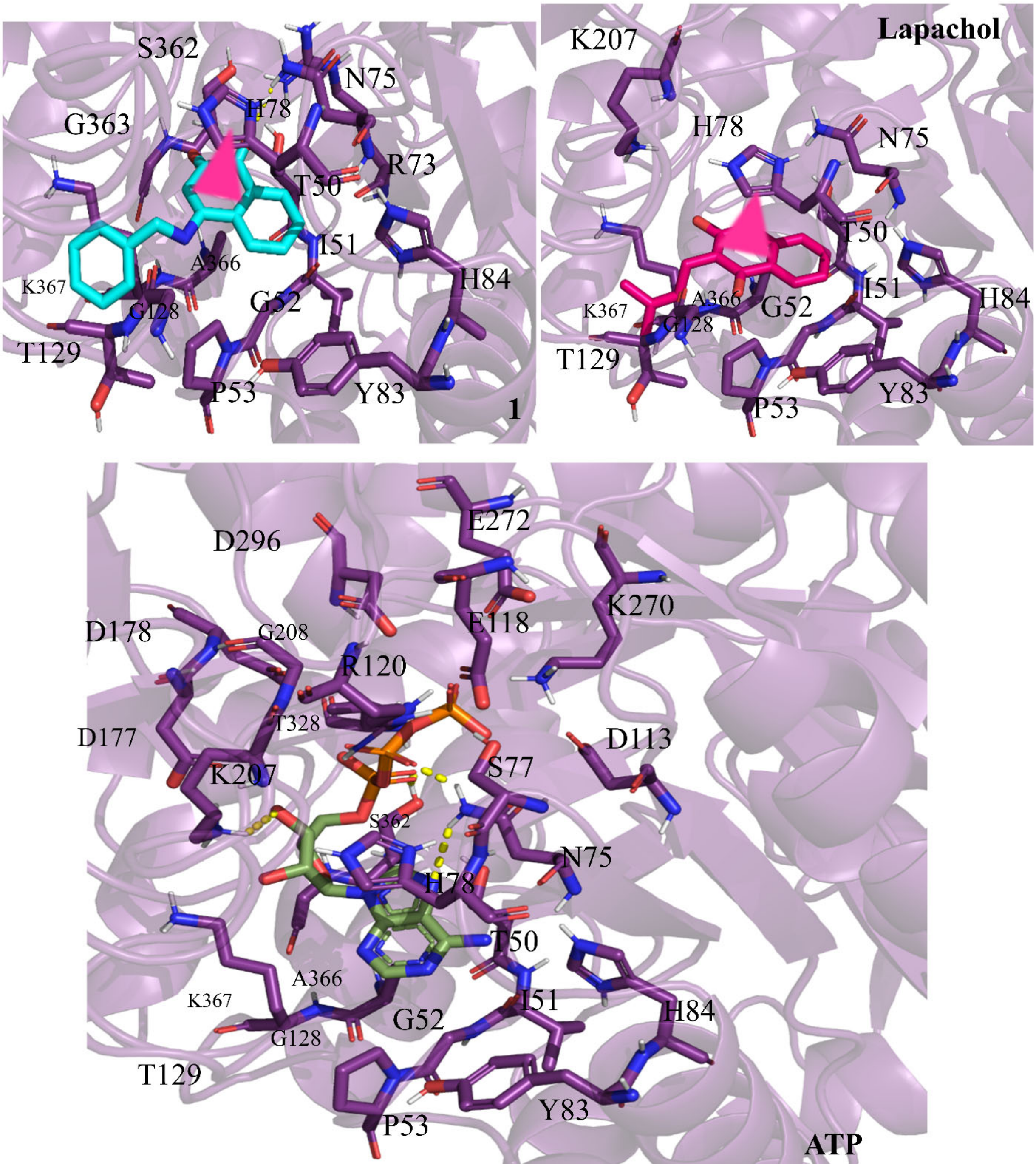

Figure S30. Molecular docking of the compound 1 (E=-9.0 kcal mol-1), the inhibitor lapachol and 7-(1H-benzimidazol-7-yl)-N-(3,4,5-trimethoxyphenyl)-1,3-benzoxazol-2-amine in the active site of RSK2. Compound 1 is represented in cyan, lapachol is represented in pink, and 7-(1H-benzimidazol-7-yl)-N-(3,4,5-trimethoxyphenyl)-1,3-benzoxazol-2-amine is represented in gold. Yellow dashes in the image represent hydrogen binding interactions.

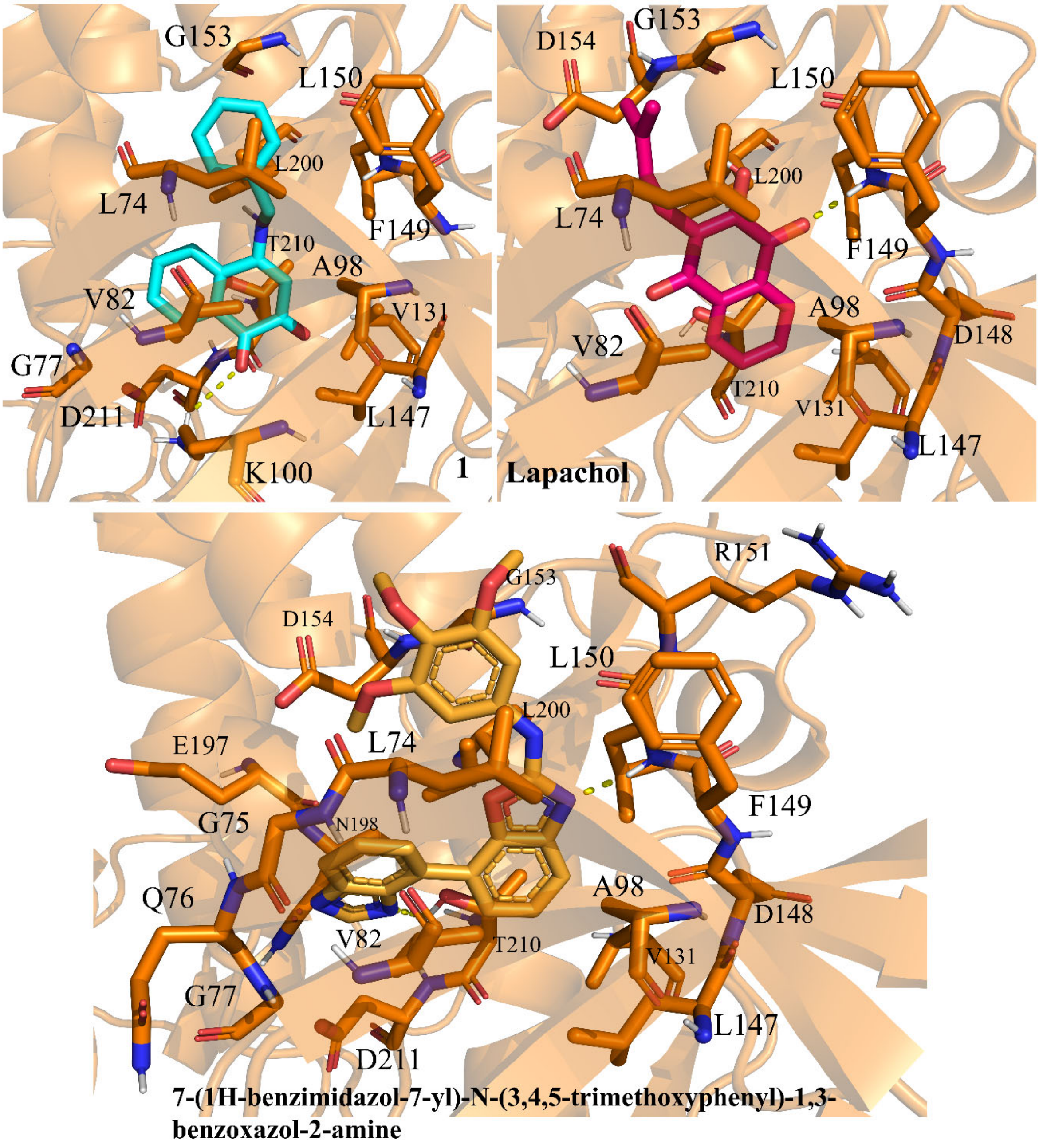

Supplement: Supplementary file 1 [file biomedicines-14-00757-s001.zip › biomedicines-4149132-supplementary.pdf]
